# Groove-Based Calibration of Multicore Fiber Bragg Grating Shape Sensors with Rotation-Angle Correction

**DOI:** 10.3390/s26196214

**Published:** 2026-09-30

**Authors:** Yi Jiang, Martin Angelmahr, Ahmad Abdalwareth, Yannick Willer, Günter Flachenecker, Wolfgang Schade

**Affiliations:** 1 Institute for Energy Research and Physical Technologies (IEPT), Clausthal University of Technology, Am Stollen 19B, 38640 Goslar, Germany; yj14@tu-clausthal.de (Y.J.);; 2 Department of Fiber Optical Sensor Systems, Fraunhofer Heinrich Hertz Institute, Am Stollen 19H, 38640 Goslar, Germanyguenter.flachenecker@hhi.fraunhofer.de (G.F.)

**Keywords:** fiber Bragg gratings, multicore fiber, shape sensing, groove-based calibration, rotation-angle correction, femtosecond-laser processing

## Abstract

**Highlights:**

- A calibration method based on grooved curvature templates is developed for MCF-FBG shape sensors.
- The effective rotation angle used for sinusoidal fitting is corrected jointly for the four FBG responses.
- A 20 cm four-core MCF containing 200 femtosecond-laser-inscribed FBGs in 50 sensor planes is demonstrated.
- Planar validation yielded tip-position errors of approximately 2–5 mm, compared with approximately 6–13 mm for conventional sine calibration.

**Abstract:**

Fiber-optic shape sensors require accurate calibration of the spatial orientation and effective core distances of their sensing planes. We present a rotation-angle correction procedure for sinusoidal calibration of multicore fiber Bragg grating (MCF-FBG) shape sensors. The method is demonstrated using a constant-radius groove template and a single rotation stage. Within the groove fixture, groove–fiber friction, finite torsional stiffness, and non-ideal boundary conditions may contribute to deviations between the local sensor-plane rotation and the motor-imposed angle, resulting in distorted sinusoidal wavelength responses and biased curvature estimation. The proposed method addresses deviations from the assumed sinusoidal response by jointly correcting the effective rotation angle assigned to the four FBG responses in each sensor plane while leaving the measured wavelength shifts unchanged. The method was demonstrated using a 20 cm four-core single-mode fiber containing 200 femtosecond-laser-inscribed FBGs arranged in 50 sensor planes at 4 mm spacing. Quantitative validation with planar constant-curvature templates showed tip-position errors of approximately 2–5 mm, compared with approximately 6–13 mm for conventional sine calibration. An additional endpoint-position test of the 50-plane sensor yielded a mean error of 11.28 mm and an RMSE of 13.73 mm, compared with 19.03 mm and 21.55 mm, respectively, for conventional sine calibration. Additional non-planar bending tests qualitatively illustrated the sensor’s three-dimensional reconstruction capability. The results show that rotation-angle correction can improve calibration accuracy while retaining a single-rotation-stage groove fixture.

## 1. Introduction

Fiber-optic shape sensing based on fiber Bragg grating (FBG) technology has been used in a wide range of applications, including robotics and medicine [1,2]. For fiber optical 3D shape detection based on FBG sensor elements, the shape recalculation is realized with the measurement of local curvatures followed by a curvature interpolation between the FBGs. The local curvatures correlate with the strains calculated from wavelength shifts or intensity changes from the shift in mode fields caused by mechanical deformation of the optical fiber. Therefore, the FBG sensor elements have to be arranged off the neutral bending axis for curvature recognition. Several different approaches such as single-mode fiber with FBG in cladding waveguide [3,4,5,6], composite structure with multi-fibers [7,8,9], emerging fiber structures [10,11], and FBGs in multicore fibers (MCF) [12,13] are reported. Among these concepts, multicore fiber Bragg grating (MCF-FBG)-based shape sensors have gained particular attention because the fixed relative positions of the cores provide an inherent geometric reference frame for reconstructing bending magnitude and direction within a single fiber. Recent MCF-based studies have demonstrated catheter and medical-device shape reconstruction [13], dense multicore interrogation schemes [14,15], femtosecond-laser-direct-written MCF sensor fabrication [14,15] and navigation of long flexible endovascular instruments [16]. These studies show that reconstruction accuracy depends not only on the optical interrogation method, but also on sensor geometry, mechanical integration, validation conditions, and calibration quality. In particular, small deviations in sensor geometry or calibration artifacts can lead to significant errors in curvature estimation and three-dimensional shape reconstruction [1,17,18]. The relevance of dedicated calibration and characterization procedures for multicore fiber-based shape sensors has also been demonstrated in previous studies. In particular, Idrisov et al. [19] showed that systematic calibration and correction steps can significantly improve curvature estimation accuracy in multicore fiber shape sensing systems, underlining calibration as a key factor for reliable three-dimensional shape reconstruction. Tan et al. [20] used a genetic-algorithm-based self-calibration to optimize relative FBG placement angles around a sensing cross-section. Zhang et al. [21] addressed azimuthal variations along helical multicore fibers using the phase–azimuth relationship of strain distributions.

Here we demonstrate a new calibration procedure based on common corrections of the effective rotation angles assigned to the four FBG responses during a constant-radius calibration sweep, from which the individual effective distances are determined.

Experimental validation was performed using a 20 cm long four-core MCF incorporating 200 equidistant FBGs. Thereby, four FBGs in a cross-section of the multicore fiber formed a so-called sensor plane, resulting in 50 sensor planes along the fiber. For each sensor plane, the local curvature was recalculated using the signals of the four corresponding FBG sensors. For accurate measurements, the sensor planes require precise calibration. A commonly used approach is the so-called sine calibration method, in which the FBG signals are recorded while the sensor is bent with a constant radius in all directions [18]. For accurate sine calibration, the local angular orientation of each sensor plane should be known throughout the rotation. In practice, this requirement is difficult to fulfill for relatively long and flexible fibers in a simple groove-based calibration setup. Groove–fiber friction, finite torsional stiffness, and mechanical constraints outside the bending template may contribute to non-ideal transfer of the imposed rotation along the fiber, so that the local rotation angle at a sensor plane may deviate from the motor-imposed angle. This deviation may appear as phase distortion in the sinusoidal wavelength responses and may result in systematic calibration errors. Within this work, a common correction of the effective rotation angle was used to fit the four FBG responses in each sensor plane, while preserving the measured wavelength shifts. The method is demonstrated with a constant-radius groove and a single rotation stage, without local angular feedback or synchronized dual-end rotation. For the 20 cm MCF-FBG sensor, planar validation yielded tip-position errors of approximately 2–5 mm after rotation-angle correction, compared with approximately 6–13 mm for conventional sine calibration.

## 2. Femtosecond Laser Inscribed FBGs in Multicore Fiber

**Figure 1:**
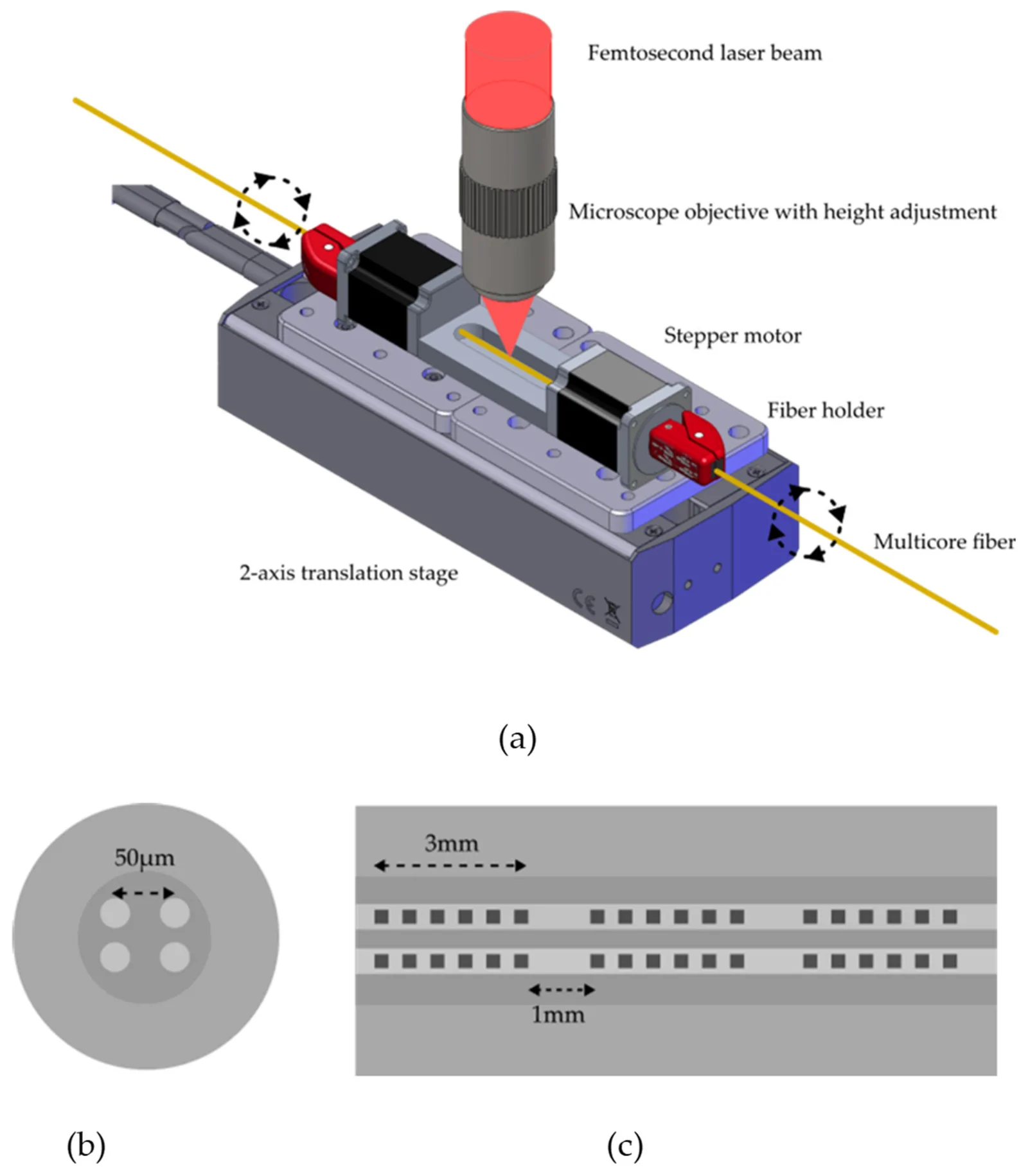
(**a**) Experimental setup of the point-by-point femtosecond laser writing system. The yellow line represents the multicore fiber, and the circular arrows indicate rotation of the fiber holders about the fiber axis. (**b**) Schematic sketch of the cross-section showing the geometric arrangement of the multicore fiber with four single-mode cores. (**c**) Schematic of 3 mm-long FBGs with 1 mm gaps, corresponding to a 4 mm center-to-center spacing between adjacent sensor planes (top view).

The experimental setup of the point-by-point femtosecond laser writing system is shown in Figure 1. A commercially available femtosecond laser source (seed laser Tsunami, amplifier Spitfire Pro F-XP; Spectra-Physics, Milpitas, CA, USA, with a center wavelength of 800 nm and pulse duration of 100 fs) is used in this setup. The laser pulses are focused into the core of a four-core optical fiber (SM-4C1500(8.0/125)/001; Fibercore, Southampton, United Kingdom) using a 40× microscope objective (LD Plan-Neofluar 40×, Zeiss, Oberkochen, Germany). The fiber is fixed by a pair of fiber holders (BFT1; Thorlabs, Newton, MA, USA), which are connected to stepper motors (PD28-1-1021-TMCL; TRINAMIC Motion Control GmbH, Hamburg, Germany). For the precise positioning of the laser focus inside the fiber, the fiber holder is mounted on top of a computer-controlled precise positioning system with two horizontally moving translation stages (N-565, Physik Instrument, Karlsruhe, Germany). The vertical position of the laser focus in the fiber core is controlled by a microscope objective mounted on a translation stage.

**Figure 2:**
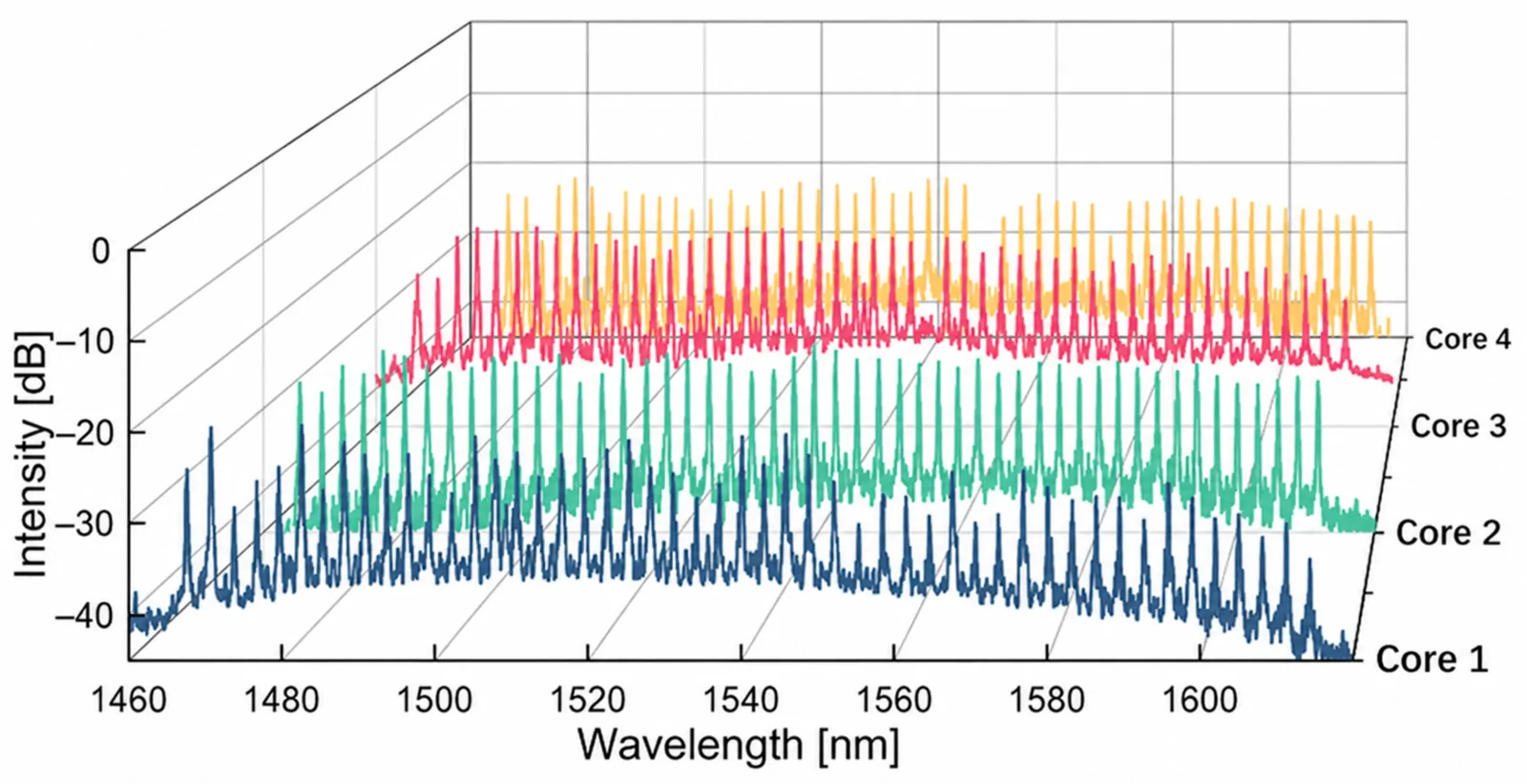
Spectrum of the fiber-optic shape sensor with 200 FBG sensors, equidistantly distributed over the four single-mode cores of a 20 cm long MCF.

For fabrication of the shape sensor, FBGs are inscribed into the four cores of a single-mode multicore fiber using point-by-point fs-laser technology. In each core, 50 FBGs with a length of 3 mm and a center-to-center spacing of 4 mm were inscribed. Four FBGs within a cross-section define one sensor plane, resulting in 50 sensor planes over a total length of 20 cm. The corresponding FBGs across the four cores were designed to have the same nominal Bragg wavelengths. The combined reflection spectra of all 200 FBGs are shown in Figure 2. Two additional sensors with 5 and 25 sensor planes were fabricated and calibrated independently. Their spectra and calibration examples are provided in Appendix C.

## 3. Curvature Recalculation and Shape Reconstruction

For the shape measurements, the presented sensor follows the conventional response of FBGs in single-mode fiber, which relates the Bragg wavelength shift ∆*λ* to strain ε and temperature variation ∆T, as expressed by Equation (1) [22]:

(1) $$\frac{∆\lambda }{\lambda }=\left(1-p_{e}\right)*\varepsilon +\left(\alpha +\frac{1}{n}\zeta \right)*∆T$$

where *p_e_* = 0.22 is the effective strain-optic coefficient used in calibration and reconstruction, *α* = 0.55 × 10^−6^ K^−1^ is the thermal expansion coefficient for fused silica [19], *n* = 1.447 is the refractive index of the multicore fiber used, and ζ is the thermo-optic coefficient.

**Figure 3:**
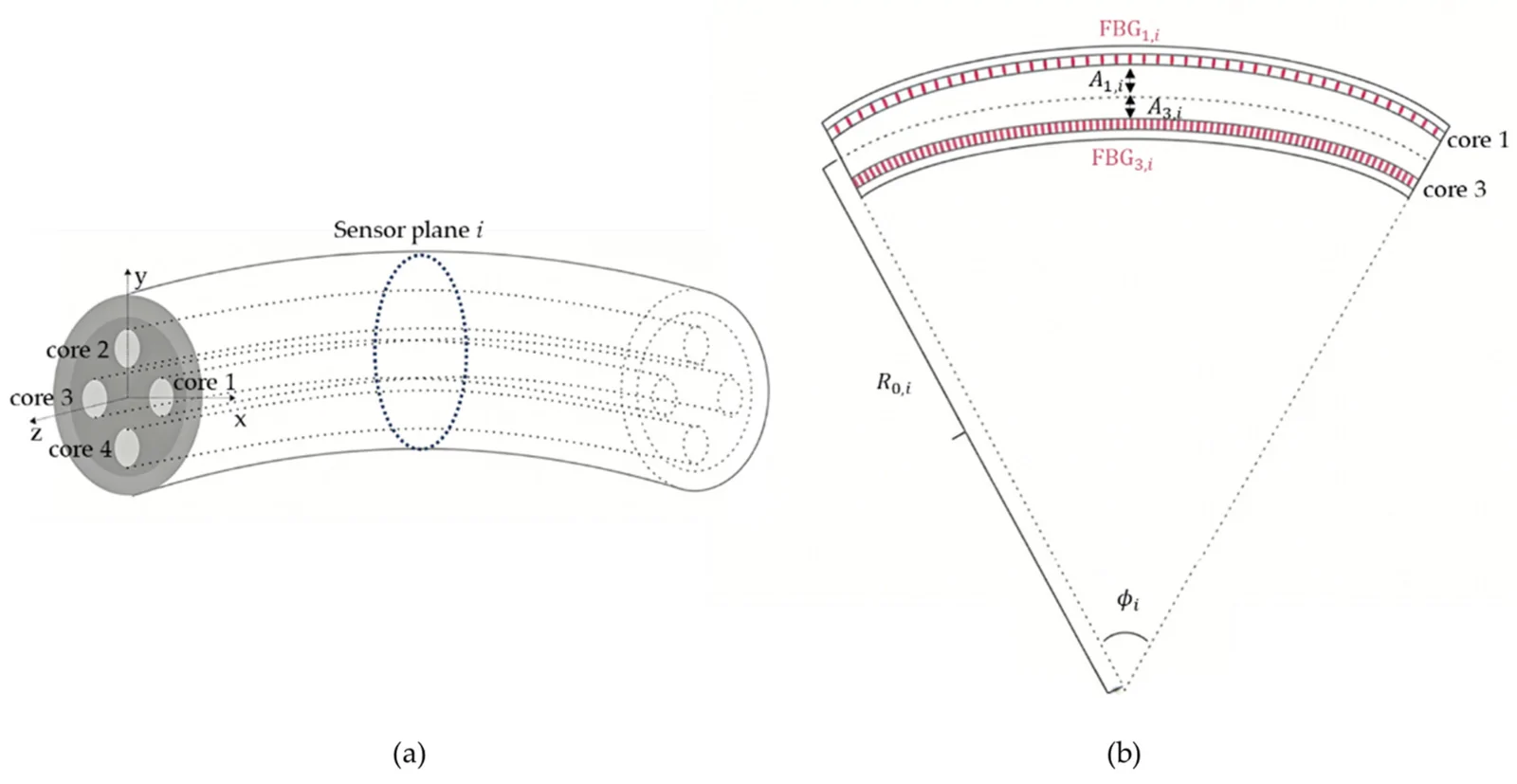
(**a**) Schematic sketch for multicore fiber during the bending process. (**b**) Geometric principle for the bending-induced strain of multicore fiber in the xz-plane of sensor plane *i*.

To reconstruct the complete fiber shape, the bending in each sensor plane *i* (with *i* = 1,…,50) must be determined (Figure 3). Within each sensor plane, the four cores are indexed by *k* ∈ {1,2,3,4}, forming the geometric basis for curvature estimation in the spatial *x*- and *y*-directions. The local bending of the fiber in the xz- and yz-planes derives from the bending-induced axial strain in x- and y- directions. For bending in the xz-plane, the averaged strain ε*_x_*_,*i*_ is obtained from the difference in the axial strains measured in the FBG of core 1 and core 3 within the sensor plane *i*:

(2) $$\varepsilon_{x,i}=\frac{1}{2}\left(\varepsilon_{1,i}-\varepsilon_{3,i}\right)$$

in which *ε*_1,*i*_ and *ε*_3,*i*_ denote the bending-induced strains of the FBGs in core 1 and core 3 within sensor plane *i*, respectively. The strain difference between FBGs in core 1 and core 3 accounts for the opposite elongation and compression of the two cores with respect to the neutral bending axis. One core undergoes tensile strain, while the FBG on the counterpart of the neutral axis undergoes compressive strain. An advantage of using two symmetrically positioned cores is the inherent compensation of uniform temperature-induced wavelength shifts. Since temperature variations affect both cores equally, their influence is eliminated in the differential strain *ε_x_*_,*i*_. Therefore, according to Equations (1) and (2) the strain in the xz- and yz-planes can be expressed as:

(3) $$\varepsilon_{x,i}=\frac{1}{2\left(1-p_{e}\right)}\left(\frac{∆\lambda_{1,i}}{\lambda_{1,i}}-\frac{∆\lambda_{3,i}}{\lambda_{3,i}}\right)$$

(4) $$\varepsilon_{y,i}=\frac{1}{2\left(1-p_{e}\right)}\left(\frac{∆\lambda_{2,i}}{\lambda_{2,i}}-\frac{∆\lambda_{4,i}}{\lambda_{4,i}}\right)$$

For a given sensor plane *i*, all four cores experience the same bending angle *ϕ_i_*. Therefore, the arc length of FBG in core *k* can be written as

*L_k_*_,*i*_ = *ϕ_i_ R_k_*_,*i*_ (5)

For bending in the xz-plane, the following distance relations apply to cores 1 and 3; the corresponding relations for cores 2 and 4 are obtained for bending in the yz-plane.

Here, *R_k_*_,*i*_ represents the bending radius of FBG in core *k* in sensor plane *i*. *R_k_*_,*i*_ can be expressed as

*R_k_*_,*i*_ = *R*_0,*i*_ ± *A_k_*_,*i*_ (6)

where *R*_0,*i*_ denotes the local bending radius of the neutral axis in sensor plane *i* and *A_k_*_,*i*_ represents the calibrated distance of FBG in Core *k* to the neutral axis in sensor plane *i*. From Equations (5) and (6), the arc length can be expressed as

*L_k_*_,*i*_ = *ϕ_i_*(*R*_0,*i*_ ± *A_k_*_,*i*_) = *ϕ_i_R*_0,*i*_ ± *ϕ_i_A_k_*_,*i*_ = *L*_0,*i*_ ± *ϕ_i_A_k_*_,*i*_ (7)

*L*_0,*i*_ = *ϕ_i_R*_0,*i*_ denotes the arc length of the neutral bending axis in sensor plane *i*. Then, the axial strain in core *k* can be expressed as

(8) $$\varepsilon_{k,i}=\frac{{(L}_{k,i}-L_{0,i})}{L_{0,i}}=\pm \frac{\phi_{i}A_{k,i}}{L_{0,i}}$$

From Equations (2) and (8), the bending-induced strain in the xz-plane yields

(9) $$\varepsilon_{x,i}=\frac{1}{2}\left(\varepsilon_{1,i}-\varepsilon_{3,i}\right)=\frac{\phi_{x,i}A_{x,i}}{L_{0,i}}$$

$A_{x,i}=\frac{1}{2}(A_{1,i}+A_{3,i})$ denotes the averaged distance to the bending neutral axis of the FBGs in cores 1 and 3. Thus, the bending angle in xz-plane can be written as

(10) $$\phi_{x,i}=\frac{\varepsilon_{x,i}L_{0,i}}{A_{x,i}}=\frac{2\varepsilon_{x,i}L_{0,i}}{\left(A_{1,i}+A_{3,i}\right)}$$

Analogously, the bending angle in the yz-plane can be expressed as

(11) $$\phi_{y,i}=\frac{\varepsilon_{y,i}L_{0,i}}{A_{y,i}}=\frac{2\varepsilon_{y,i}L_{0,i}}{\left(A_{2,i}+A_{4,i}\right)}$$

The corresponding bending radii are obtained as

(12) $$R_{x,i}=\frac{L_{0,i}}{\phi_{x,i}}=\frac{A_{x,i}}{\varepsilon_{x,i}}=\frac{A_{1,i}+A_{3,i}}{2\varepsilon_{x,i}}$$

(13) $$R_{y,i}=\frac{L_{0,i}}{\phi_{y,i}}=\frac{A_{y,i}}{\varepsilon_{y,i}}=\frac{A_{2,i}+A_{4,i}}{2\varepsilon_{y,i}}$$

Therefore, the bending direction within sensor plane *i* is defined by

*β_i_* = atan2(*ϕ_y_*_,*i*_,*ϕ_x_*_,*i*_) (14)

The local curvature components are *κ_x_*_,*i*_ = *ε_x_*_,*i*_/*A_x_*_,*i*_ and *κ_y_*_,*i*_ = *ε_y_*_,*i*_/*A_y_*_,*i*_. Cubic-spline interpolation provides the curvature between the 50 equidistant sensor planes. Starting from the prescribed base position and orientation, reconstruction proceeds along the fiber. The local curvature and bending direction determine the bending of each successive segment. The endpoint position and orientation of one segment provide the starting conditions for the next. Joining these segments gives the reconstructed centerline and tip position. Axial fiber twist is not independently measured or compensated.

## 4. Groove-Based Calibration Procedure and Rotation-Angle Correction

The four-core arrangement provides the nominal geometry for curvature reconstruction. The effective FBG-to-neutral-axis distances *A_k,i_* are determined from the fitted single-FBG wavelength amplitudes at the known template radius using Equation (19). The mean effective distances in the two orthogonal directions are obtained using Equation (20) and combined with the differential strains from Equations (3) and (4) to reconstruct bending radii according to Equations (12) and (13). These calibrated quantities are response-based effective distances, rather than independent measurements of the physical core positions.

**Figure 4:**
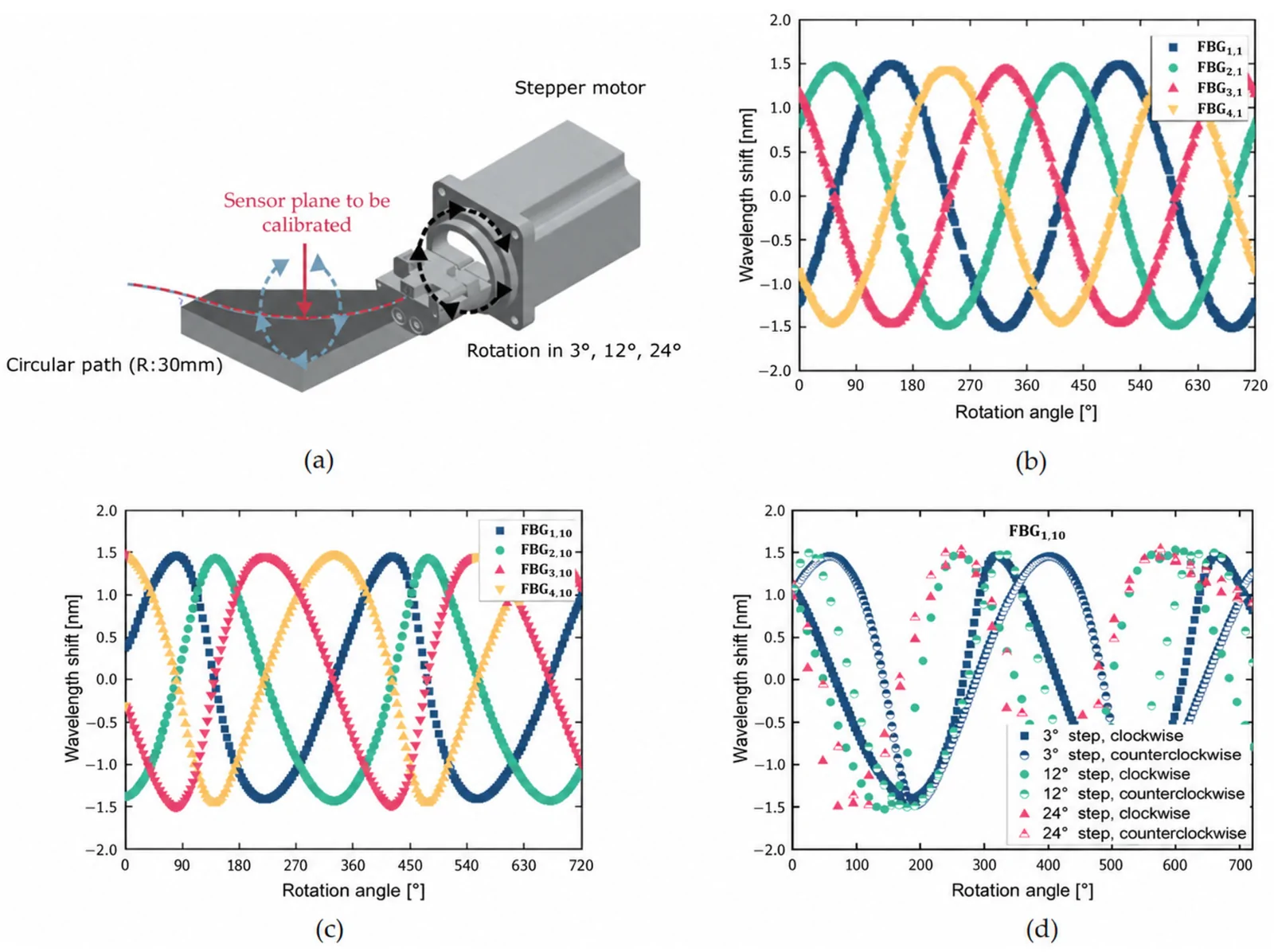
(**a**) Single-rotation-stage groove fixture used for sine calibration. The curved arrows schematically indicate rotation of the fiber about its longitudinal axis during calibration. (**b**) Sinusoidal responses of sensor plane 1 under conventional sine calibration. (**c**) Distorted responses of sensor plane 10. (**d**) Uncorrected response of FBG_1,10_ under clockwise and counterclockwise rotation with step sizes of 3°, 12° and 24°.

For the calibration procedure, the sensor fiber was inserted into a template with a 30 mm circular-radius groove, as shown in Figure 4a. The template kept the fiber in a defined bending position throughout the experiment. The fiber was rotated around its own axis inside the template’s groove using a rotation stage (SM1F1-250; Thorlabs) driven by a stepper motor (PD28-1-1021-TMCL; TRINAMIC Motion Control GmbH), where it was only fixed to the rotation stage. For each rotation step, the wavelength shifts in the four FBGs within one sensor plane were recorded with a commercial optical sensing instrument (si155 interrogator; Micron Optics, Atlanta, GA, USA). A centroid peak recognition was applied to obtain the FBG wavelengths.

When the fiber was rotated inside the calibration template, it underwent circular bending with a constant curvature determined by the template radius *R*_tem_. During the rotation, a short free-standing fiber end was used behind the template to reduce torsional constraint. However, friction inside the template during fiber rotation may contribute to inhomogeneous torsion (see Figure 4a). Consequently, the effective rotation experienced by a given sensor plane may deviate from the motor-imposed angle. Such deviations can result in phase distortions of the measured sinusoidal signals and systematic deviations from the ideal sinusoidal response. Therefore, these effects may introduce an additional systematic error during calibration. Local sensor-plane rotation was not measured independently; the proposed mechanical explanation therefore does not exclude contributions from intrinsic twist, core geometry or optical effects.

It is therefore useful to distinguish between the motor-imposed rotation angle and the local angular orientation of a given sensor plane. A constant angular offset can be absorbed by the fitted phase term in the sine model. By contrast, an angle-dependent or direction-dependent local angular deviation cannot be represented by a single-phase parameter and appears as the distorted sinusoidal response shown in Figure 4c. Possible hardware approaches include synchronized dual-end rotation and local angular feedback. Their effectiveness, calibration time, and hardware cost were not compared quantitatively in this work. The proposed method corrects the effective rotation angle used for sinusoidal fitting while retaining calibration in a single-rotation-stage groove setup.

The bending and rotation of the fiber inside the circular groove induce different strains for all FBGs within a sensor plane, causing phase-shifted sinusoidal changes in the FBG wavelengths from the angle of rotation (shown in Figure 4b). Within our calibration procedure, every sensor plane is calibrated separately by positioning each in the middle of the rotating template. However, in some cases, the FBG signals recorded during the calibration do not follow the assumed sinusoidal behavior well. For example, a signal distortion of the sinusoidal strain was observed from the calibration of the tenth sensor plane (see Figure 4c). The distortion is consistent with non-ideal transfer of rotation, potentially influenced by groove–fiber contact and external boundary conditions. Because local rotation was not measured independently, this interpretation does not isolate the cause of the distortion. Different rotation directions (clockwise and counterclockwise) and step sizes (3°, 12°, and 24°) were recorded to illustrate the dependence of the uncorrected response on the calibration conditions. The signals from FBG1,10 within sensor plane 10 show that the signal distortions cannot be neglected and may affect sinusoidal fitting and bias the fitted amplitudes and the effective distances derived from them (see Figure 4d).

At the known template radius, the sinusoidal wavelength amplitude of each FBG is used to determine its effective distance from the bending neutral axis. Distortion of the response against the recorded rotation angle can bias the fitted amplitude and, consequently, the calibrated distance. The rotation-angle correction therefore adjusts the rotation angles used for fitting. The offset-subtracted responses of the four FBGs are described by the following sinusoidal model, expressed in terms of the effective rotation angle *θ*:

Δ*λ*_1,*i*_ = *a*_1,*i*_
*sin*(*θ* + *φ_i_*) (15)

(16) $${\Delta \lambda }_{2,i}=a_{2,i}\sin\left(\theta +\frac{\pi }{2}+\varphi_{i}\right)$$

Δ*λ*_3,*i*_ = *a*_3,*i*_
*sin*(*θ* + *π* + *φ_i_*) (17)

(18) $${\Delta \lambda }_{4,i}=a_{4,i}\sin\left(\theta +\frac{3\pi }{2}+\varphi_{i}\right)$$

Here, *a_k_*,*_i_* is the sinusoidal wavelength amplitude of core *k* in sensor plane *i*, and *φ_i_* is the constant orientation phase offset of that sensor plane.

The following correction procedure aims to improve the sinusoidal amplitudes used for calibration when the sensor plane rotates unevenly relative to the recorded rotation angle. It adjusts the rotation angles shared by the four FBG responses while keeping all measured wavelength shifts unchanged. The procedure consists of an initial fit, a local angle correction and a final fit.

First, the four responses are fitted against the recorded rotation angle *θ*_rec_ using the Levenberg–Marquardt algorithm. The fit determines the response amplitudes, offsets and phase, together with a common angular scale *ω*. For the offset-subtracted responses, Equations (15)–(18) can be written as ${\Delta \lambda }_{k,i}=a_{k,i}\sin\left(\omega \theta_{\mathrm{rec}}+\frac{(k-1)\pi }{2}+\varphi_{i}\right)$, for *k* = 1, …, 4, with *θ* = *ωθ*_rec_. The angular quantities in this equation are expressed in radians. This angular scale describes the overall variation in the responses over the scan and is held fixed during the subsequent angle correction. Local irregularities that cannot be represented by this single scale are addressed in the next step.

Second, a hill-climbing procedure moves a window along the recorded rotation sweep of one sensor plane. The window contains consecutive angular sample positions, each with one measured value from each of the four cores. At each window position, the procedure tests a shared leftward or rightward shift in these angles; all measured wavelength shifts remain unchanged. Each trial that satisfies the correction limits and angular-order requirement is refitted with the angular scale held fixed. The current result and the two trial results are compared using the total squared fitting error over the complete four-core responses. An update is accepted only if the error reduction exceeds the specified tolerance.

The search starts with a window spanning approximately one quarter of the initially fitted period. At each window width, repeated scans use progressively smaller trial shifts. After this search is complete, the window is halved and the trial shifts restart from their largest size, retaining the corrections already accepted. This process continues down to a single sample position, where the angles assigned to the four measured core responses are adjusted together. Appendix A gives the scan sequence, numerical constraints and stopping criteria.

In the final step, the corrected rotation-angle values replace the recorded angles in the scaled model given above and are held fixed for the final sinusoidal fit. The resulting amplitudes are used in Equation (19) to calculate the effective FBG-to-neutral-axis distances. The practical benefit of this calibration is evaluated through the subsequent bending-radius and shape-reconstruction measurements.

Groove–fiber friction, non-uniform torsional transfer and intermittent motion can cause the sensor plane to follow the imposed rotation unevenly. Twisting may also be accompanied by polarization-dependent changes in the optical response. The procedure compensates for the combined influence of these effects insofar as their contribution to the response distortion can be represented by a common angular adjustment. It does not require their individual contributions to be separated. The corrected angle is an effective rotation angle used for sinusoidal fitting, not an independently measured local rotation angle; its physical interpretation and limitations are discussed in Section 6.2.

**Figure 5:**
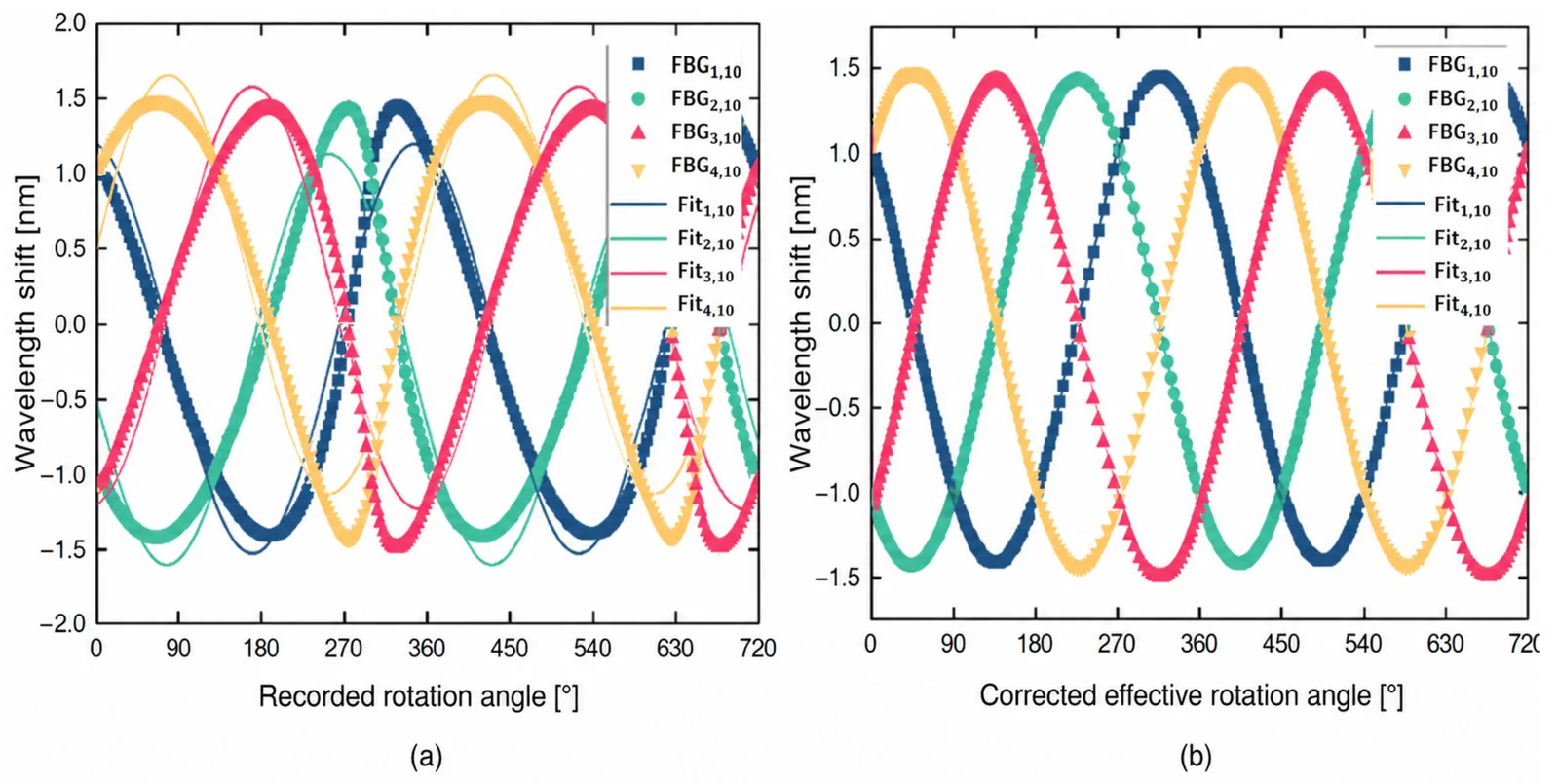
Responses of sensor plane 10 and their sinusoidal fits using (**a**) conventional sine calibration and (**b**) rotation-angle correction. The same measured wavelength shifts are plotted against the recorded rotation angle in (**a**) and the corrected effective rotation angle in (**b**).

Figure 5 illustrates the procedure for sensor plane 10 using calibration data acquired under clockwise rotation with a step size of 3°. The same measured wavelength shifts are fitted using the recorded angles before correction and the corrected angles afterwards. The coefficients of determination (R^2^) increase from 0.9517–0.9576 to 0.9998–0.9999 (Table 1). All calibration parameters used in the subsequent bending-radius and shape-reconstruction experiments reported in this work were obtained by calibrating each sensor plane individually under this same rotation condition.

For calibration of the corrected distances of FBGs to the bending neutral axis, we use the amplitudes *a_k_*_,*i*_ from the sinusoidal fit, which correspond to the maximum wavelength shifts induced by bending. Here, *a_k_*_,*i*_ is the single-FBG sinusoidal amplitude, equal to half the peak-to-peak wavelength excursion. Using $a_{k,i}=\left(1-p_{e}\right)\lambda_{k,i}\varepsilon_{k,i}^{\max}$ and $\varepsilon_{k,i}^{\max}=A_{k,i}/R_{\mathrm{tem}}$ gives

(19) Ak,i=εk,imaxRtem=Rtem(1−pe)ak,iλk,i,with k=1,…,4.

Here, *λ_k_,_i_* is the reference Bragg wavelength of the individual FBG. Each *A_k_,_i_* is an individual effective distance to the neutral bending axis, rather than an opposite-core separation. The averages used for the two orthogonal curvature components are defined in Equation (20).

Thus, the calibrated distance of each FBG in an individual core to the neutral bending axis can be determined directly from the fitted amplitude. For subsequent curvature reconstruction in Section 5, the averaged effective distances in the orthogonal directions are calculated as

(20) $$A_{x,i}=\frac{1}{2}\left(A_{1,i}+A_{3,i}\right)\mathrm{and}A_{y,i}=\frac{1}{2}\left(A_{2,i}+A_{4,i}\right)$$

**Figure A2:**
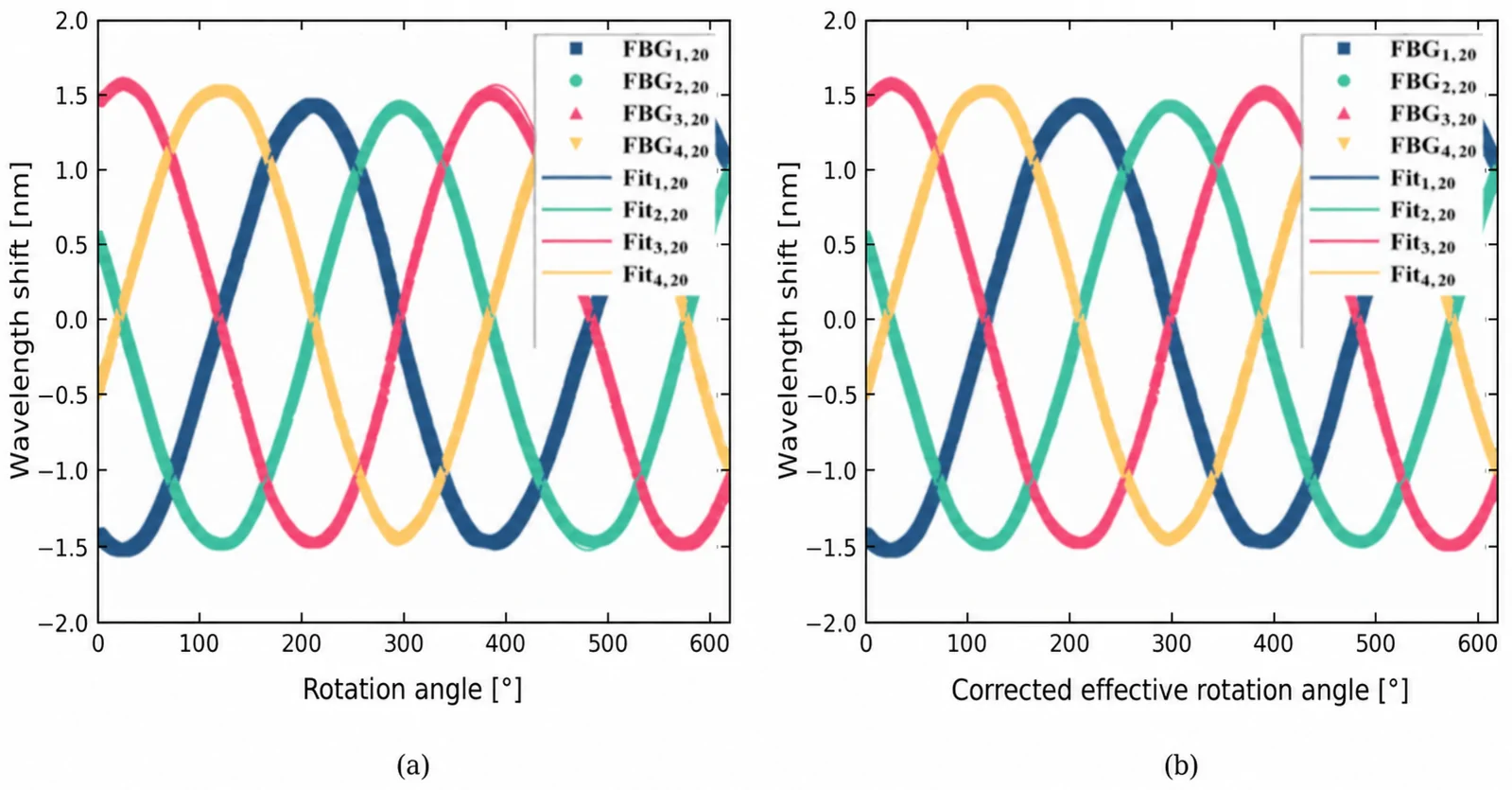
Responses of sensor plane 20 and their sinusoidal fits using (**a**) conventional sine calibration and (**b**) rotation-angle correction.

**Figure A3:**
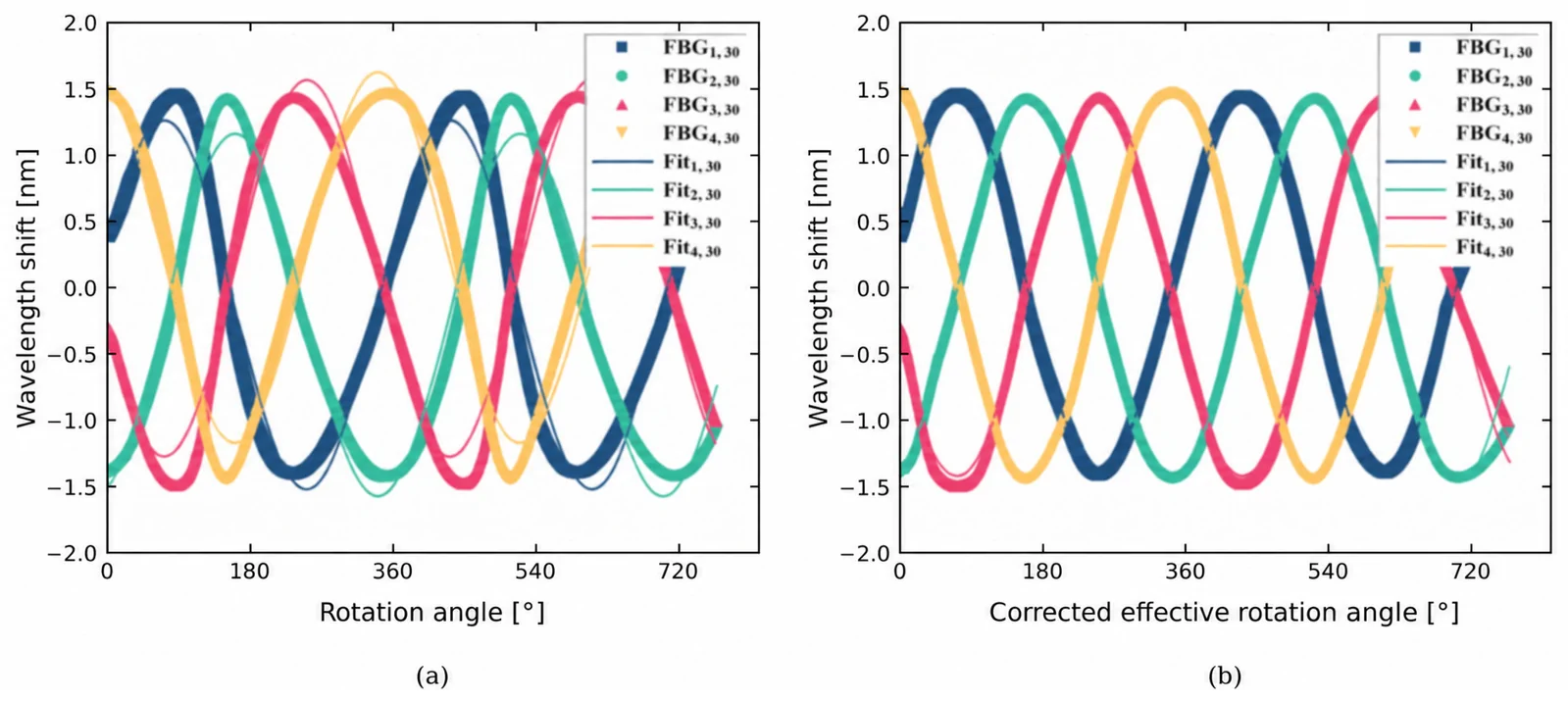
Responses of sensor plane 30 and their sinusoidal fits using (**a**) conventional sine calibration and (**b**) rotation-angle correction.

**Figure A4:**
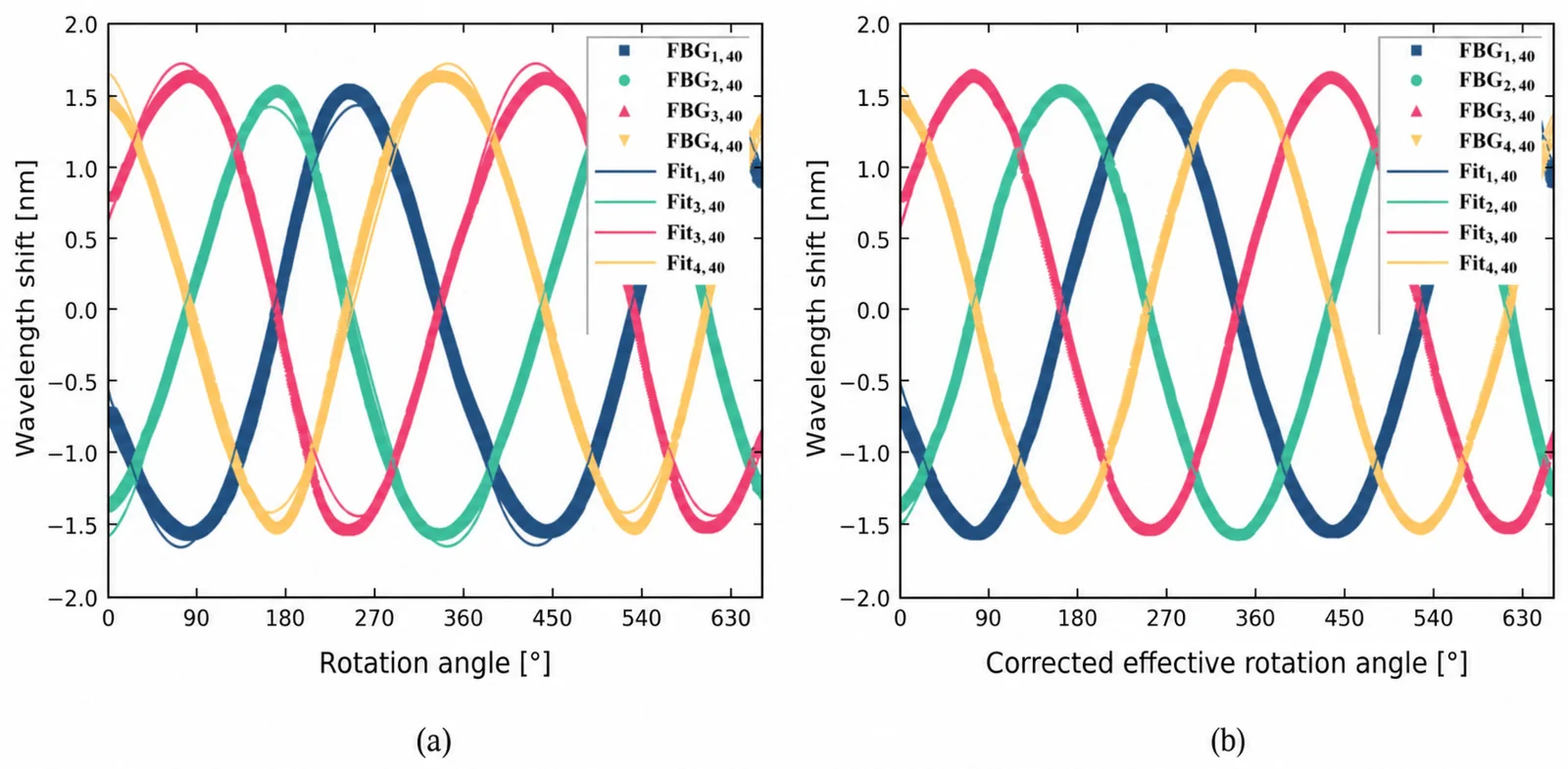
Responses of sensor plane 40 and their sinusoidal fits using (**a**) conventional sine calibration and (**b**) rotation-angle correction.

**Figure A5:**
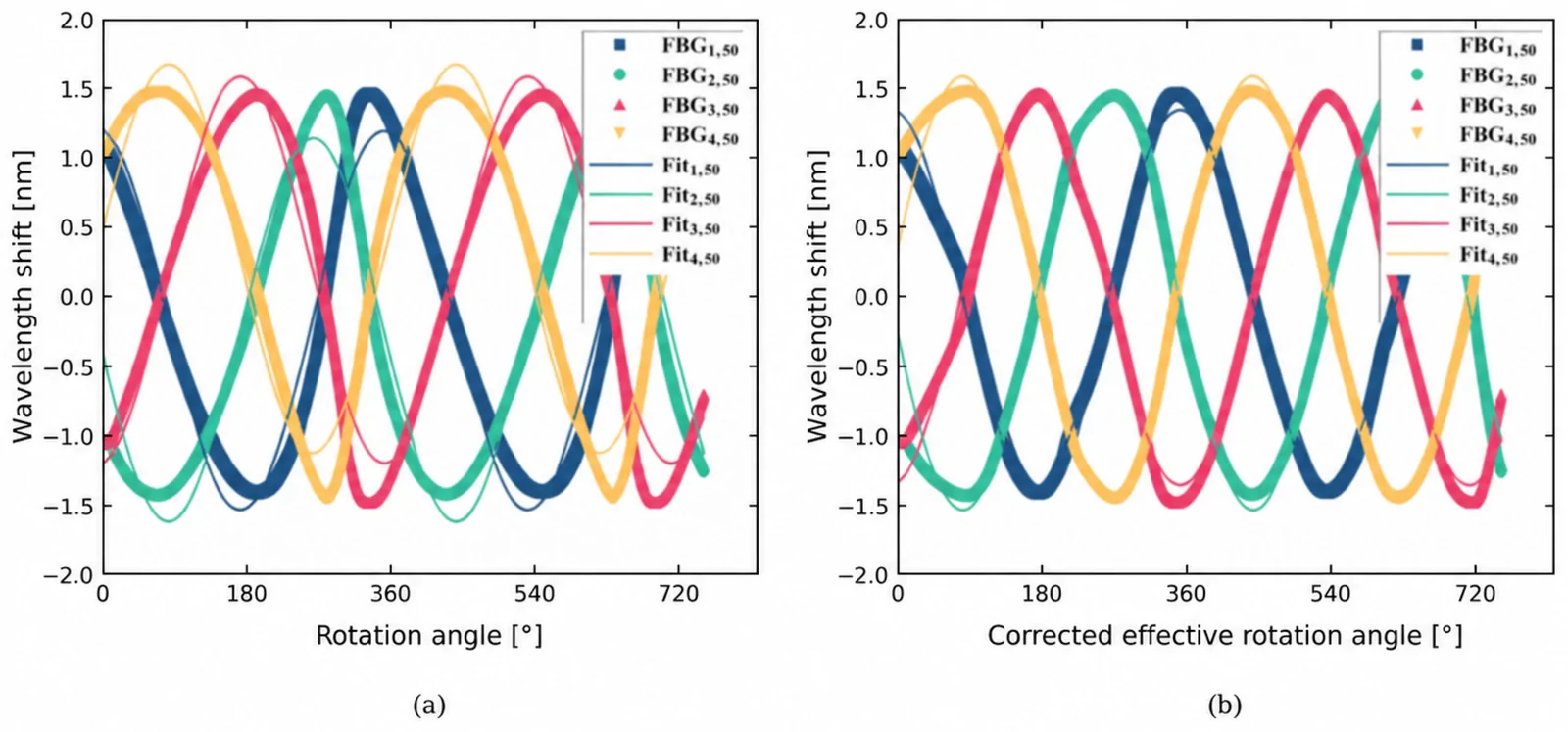
Responses of sensor plane 50 and their sinusoidal fits using (**a**) conventional sine calibration and (**b**) rotation-angle correction.

In addition to sensor plane 10 shown in Figure 5, Figure A2, Figure A3, Figure A4 and Figure A5 provide fitting examples for sensor planes 20, 30, 40 and 50. Table A1 summarizes the before/after R^2^ statistics for all 50 sensor planes, and Table A2 summarizes the effective distances of each core across the array. Across all 200 FBG responses, the mean R^2^ increases from 0.98519 ± 0.01286 to 0.99815 ± 0.00232, where SD describes the variation across responses. Sensor plane 10 is shown as a detailed example because it clearly illustrates the correction behavior observed across the array, while Table A1 provides the full distribution of fitting performance for all 50 sensor planes. These fitting statistics do not establish physical angular error or full-array bending-radius accuracy.

**Figure 6:**
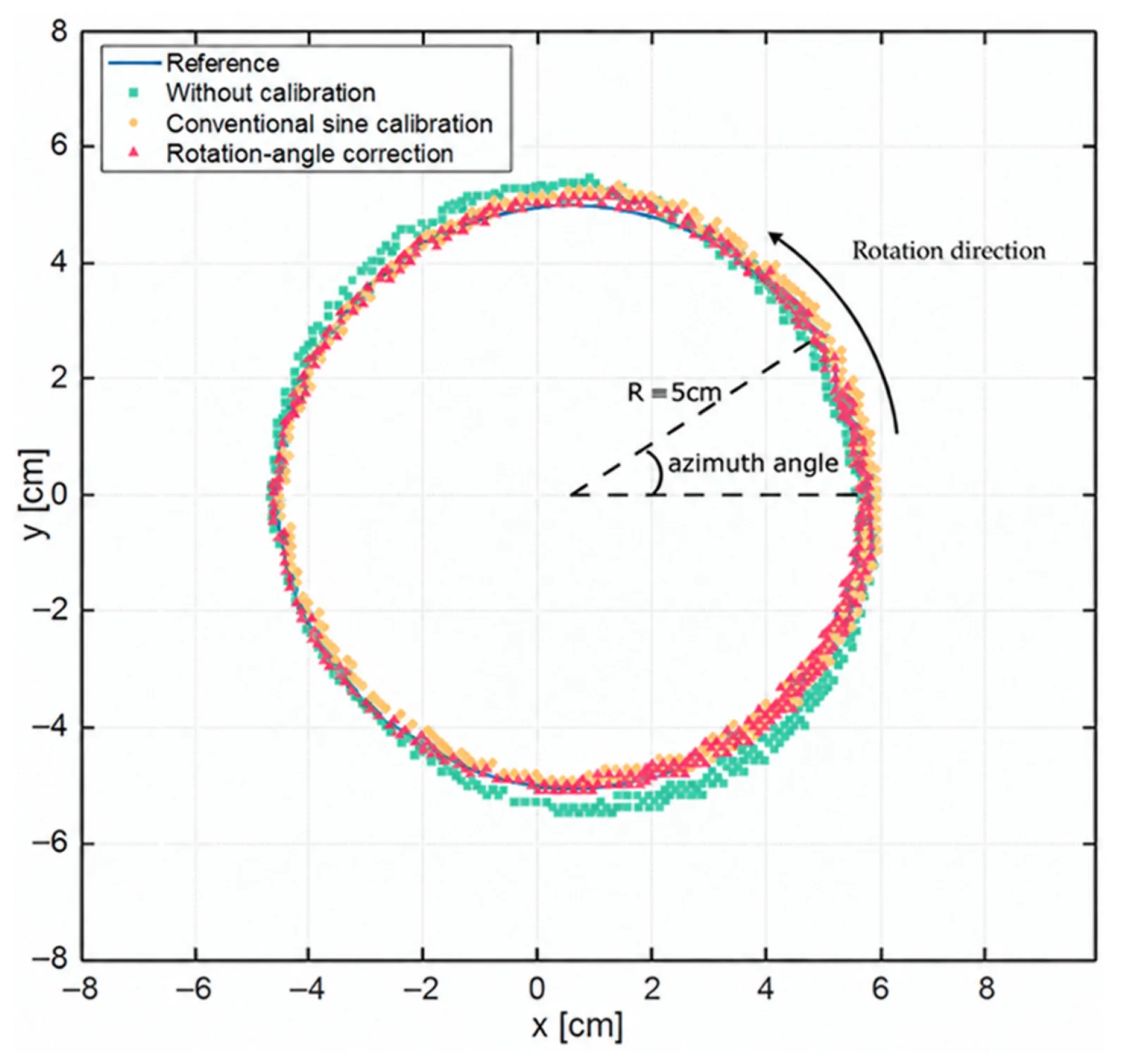
Reconstructed bending radii of sensor plane 10 at different bending directions, obtained without calibration, with conventional sine calibration and with rotation-angle correction. The reference circle has a radius of 50 mm.

To evaluate the calibration methods, sensor plane 10 was tested in a separate circular template with a radius of 50 mm while the fiber was rotated through 720°. The reconstructed bending radius was compared with the 50 mm reference across the sampled bending directions. The mean bending-radius error was 2.7 mm without calibration, 1.9 mm with conventional sine calibration and 0.7 mm with rotation-angle correction. Figure 6 shows the reconstructed radii for the three calibration methods together with the reference circle.

## 5. Shape Reconstruction and Assessment

### 5.1. Quantitative Validation Using Planar Constant-Curvature Templates

**Figure 7:**
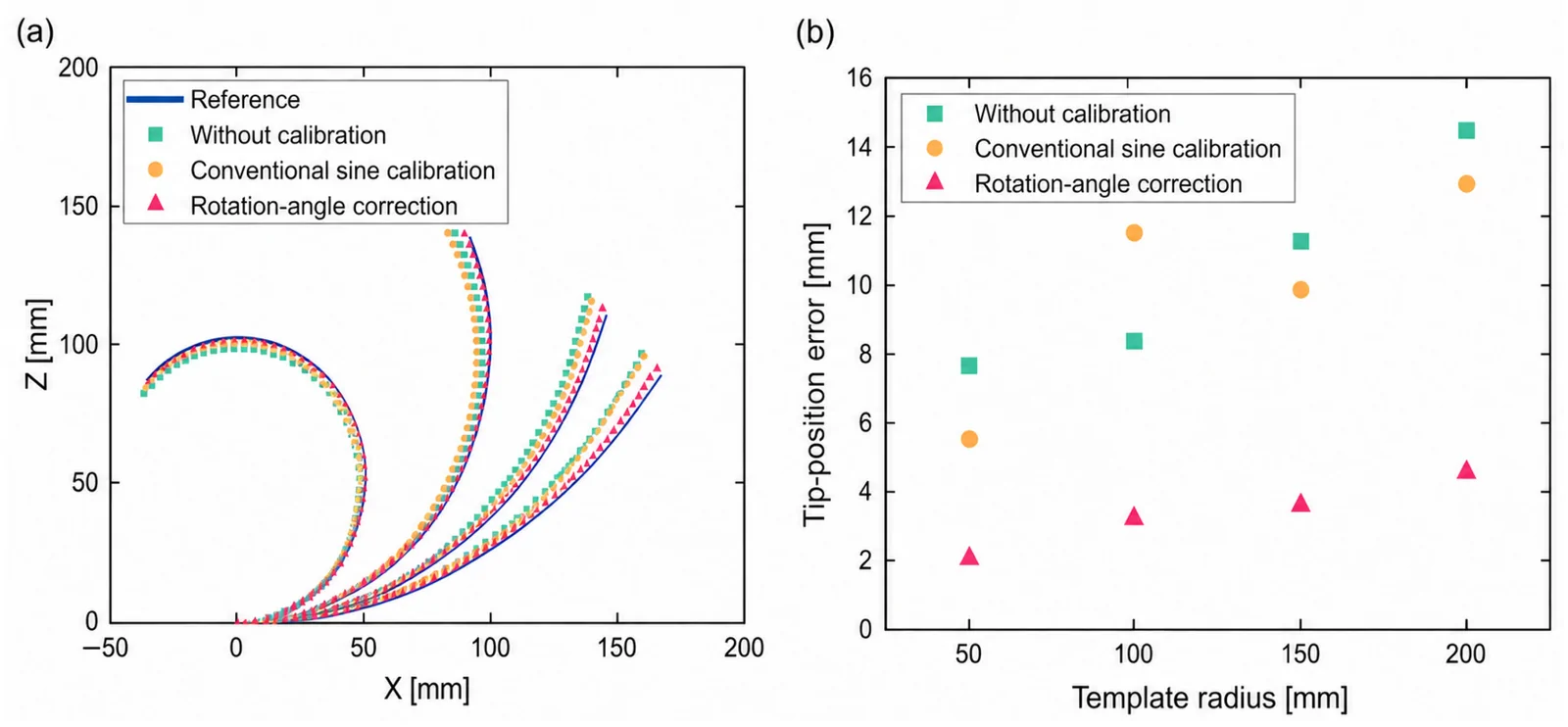
(**a**) Mean planar constant-curvature reconstructions for template radii of 50, 100, 150, and 200 mm obtained without calibration, with conventional sine calibration, and with rotation-angle correction. (**b**) Corresponding mean tip-position errors from five repeated placements.

For quantitative evaluation, the fiber optical shape sensor was placed five times in each of four planar templates with known radii of 50, 100, 150 and 200 mm. The reconstructed planar constant-curvature shapes were then compared with the corresponding template geometries. In addition to the local curvature of each sensor plane, the tip position of the sensor was recalculated. Across the four template radii, rotation-angle correction reduced the mean tip-position error to approximately 2–5 mm, compared with approximately 6–13 mm for conventional sine calibration and approximately 8–15 mm without calibration (Figure 7).

### 5.2. Qualitative Demonstration of Non-Planar Shape Reconstruction

**Figure 8:**
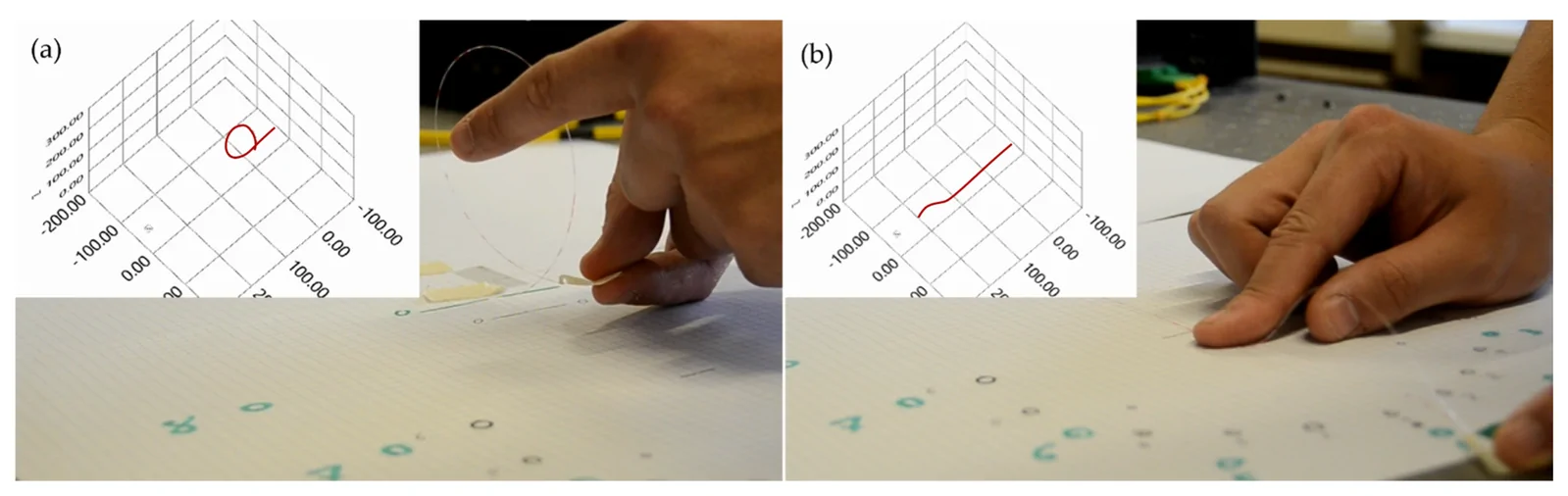
Qualitative 3D bending demonstration of the MCF-FBG shape sensor. The photographs show representative non-planar configurations, and the insets show the corresponding reconstructed 3D centerlines.

To illustrate the three-dimensional reconstruction capability beyond the planar template experiments, the sensor was additionally arranged in representative non-planar bending configurations. Since no calibrated 3D ground-truth system was available for these tests, the results are presented as a qualitative demonstration (see Figure 8).

### 5.3. Quantitative Endpoint-Position Assessment

**Figure 9:**
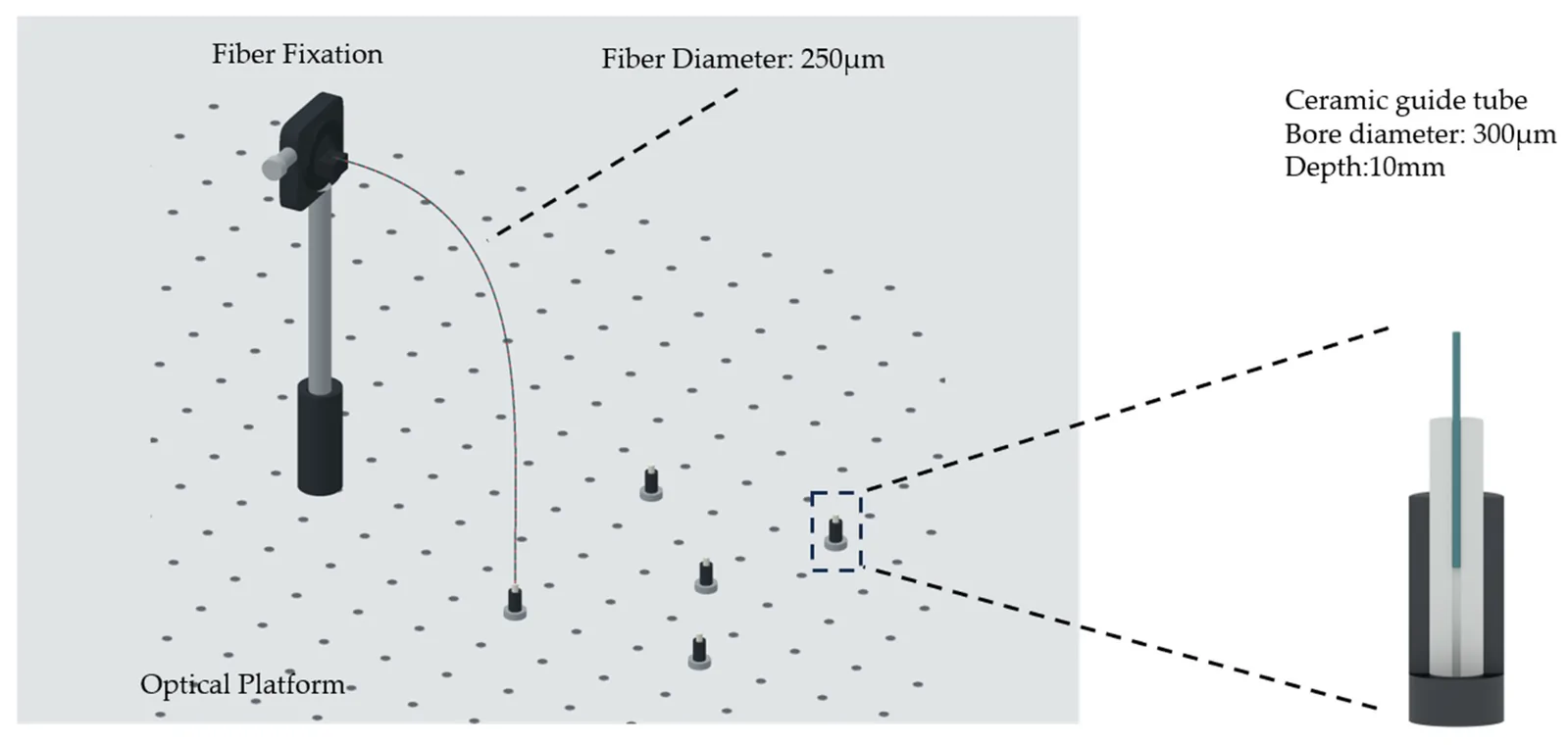
Schematic illustration of the experimental setup for endpoint-position measurements, showing the fiber fixation above the optical platform and the ceramic guide tubes at the target positions. The enlarged cutaway view shows the 250 μm-diameter fiber inserted into a guide tube with a bore diameter of 300 μm and a depth of 10 mm. The enlarged view is not drawn to scale.

**Figure 10:**
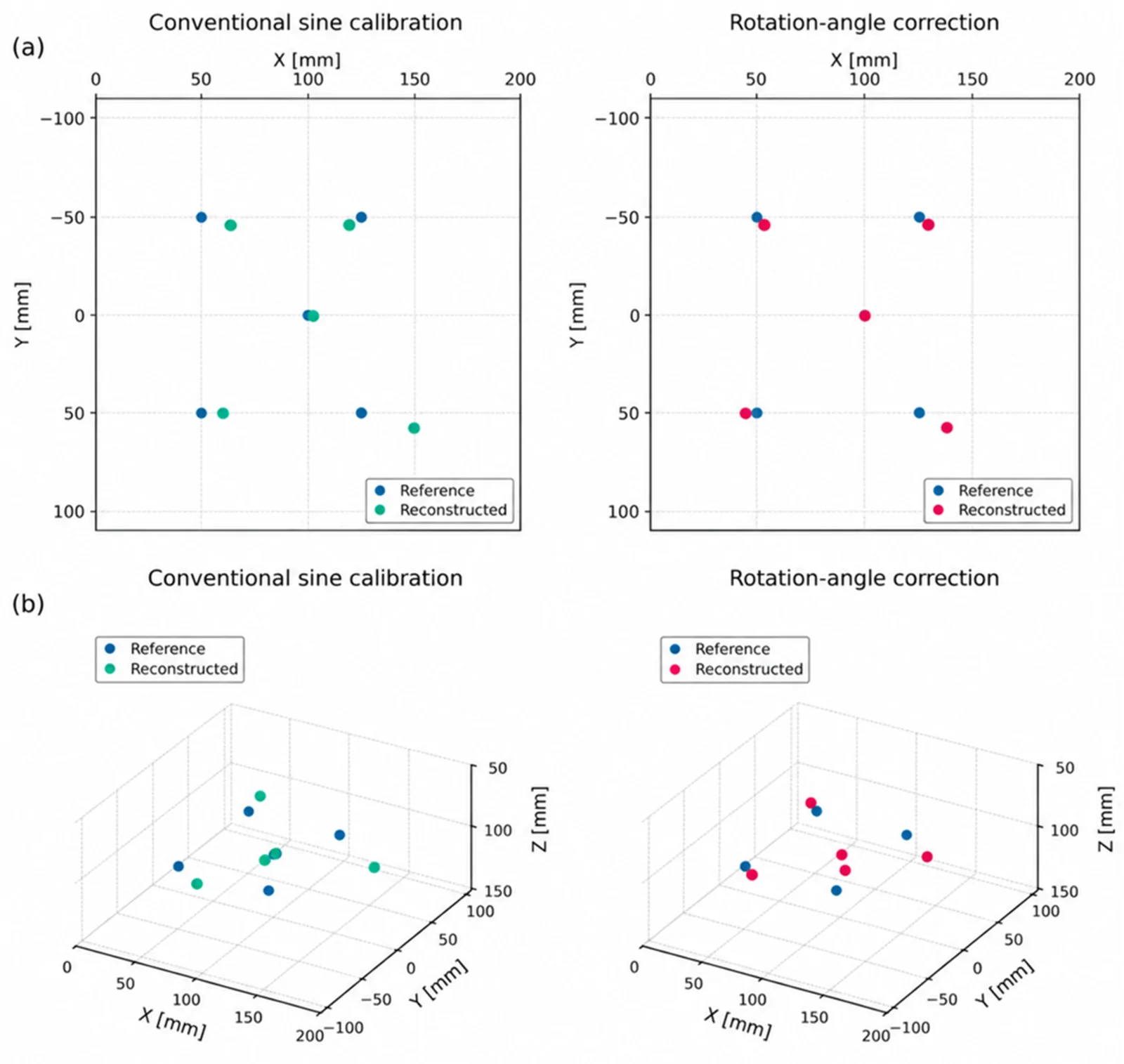
Endpoint-position assessment of the 50-plane sensor: (**a**) XY projection; (**b**) oblique XYZ view. Left: conventional sine calibration (green); right: rotation-angle correction (red); blue: reference targets.

An endpoint-position assessment was performed with the 50-plane sensor using the setup illustrated schematically in Figure 9. The reconstructed and reference endpoint positions are shown in Figure 10. The fiber base was fixed above an optical platform, with the base position and initial orientation prescribed for reconstruction. At each target, the 250 μm-diameter fiber was inserted into a ceramic guide tube with a bore diameter of 300 μm and a depth of 10 mm. The opening constrained lateral position, and the controlled insertion depth defined the endpoint position along the guide. Five endpoint records were acquired at each of five target positions for each calibration method (*n* = 25 per method).

Endpoint error was calculated as the Euclidean distance between the reconstructed and reference endpoint coordinates. Table 2 reports the mean error, RMSE, sample standard deviation SD(*n* − 1), minimum, and maximum for both calibration methods.

In the 50-plane endpoint test, rotation-angle correction reduced the mean endpoint error from 19.03 mm to 11.28 mm and the RMSE from 21.55 mm to 13.73 mm relative to conventional sine calibration. The reference targets in this test are coplanar. The measurements therefore quantify endpoint-position agreement in three-dimensional coordinates, not non-coplanar volumetric accuracy or accuracy along the complete 3D centerline.

## 6. Discussion and Limitations

### 6.1. Comparison with Previous MCF-Based Shape-Sensing Studies

Table 3 compares the proposed sensor and calibration method with representative MCF-based shape-sensing studies. As the reported error metrics vary across the literature—including curvature error, tip-position error, maximum Euclidean reconstruction error, and error per unit length—the original metrics are retained for each study to avoid misleading direct comparisons.

The correction variables differ: Tan et al. [20] optimize relative FBG placement angles, Zhang et al. [21] calibrate inter-core azimuthal relationships along a helical fiber, whereas the present method applies a common correction to the effective rotation angle used for sinusoidal fitting within each constant-radius calibration sweep.

### 6.2. Limitations

The proposed method improves calibration accuracy under non-ideal rotational conditions, but several limitations remain. The mechanical boundary conditions addressed in this work may contribute to the observed distortions. The proposed calibration procedure improves agreement with the sinusoidal model through a common correction of the effective rotation angle used for fitting, but it does not eliminate possible mechanical contributors, such as groove–fiber friction, finite torsional stiffness, stick–slip effects, or deformation of the free fiber section. The corrected effective angles describe the angular progression used for fitting, rather than an independently verified local rotation history. Synchronized dual-end rotation, low-friction guiding structures and closed-loop angular control are possible approaches to reduce these effects, but the effectiveness of these approaches was not evaluated in this work. Combining such hardware approaches with the proposed calibration procedure may further improve the overall accuracy of FBG-based 3D shape sensing.

Point-by-point femtosecond-laser-inscribed FBGs may exhibit birefringence and polarization-dependent spectral behavior, which can increase peak-tracking uncertainty, especially at large bending radii where the bending-induced wavelength shift is small [23].

While the differential evaluation of opposite cores suppresses uniform temperature-induced wavelength shifts, transverse temperature gradients, non-uniform heating along the fiber, and temperature drift during sequential calibration are not fully compensated.

The correction may fail or bias the calibrated parameters when peak-tracking errors, polarization effects, temperature gradients or non-uniform strain cannot be represented by a common effective rotation-angle correction. Each fabricated sensor and sensing plane requires individual calibration; calibrated parameters are not transferred between sensors. The additional samples demonstrate application of the procedure but do not establish manufacturing reproducibility or robustness to temperature and polarization variations.

Quantitative validation in this study is limited to planar-template tests and endpoint-position assessments with coplanar reference targets. Full-centerline non-planar accuracy and axial twist compensation were not independently evaluated.

## 7. Conclusions

This work presents a rotation-angle correction applied jointly to the four FBG responses within each plane of an MCF-FBG shape sensor. The method corrects the effective rotation angle used for sinusoidal fitting during calibration while preserving the measured wavelength shifts and is demonstrated using a constant-radius groove and a single rotation stage. In planar constant-curvature tests, tip-position errors were approximately 2–5 mm, compared with approximately 6–13 mm for conventional sine calibration. The additional endpoint test with coplanar reference targets yielded a mean error of 11.28 mm for the 50-plane sensor, compared with 19.03 mm for conventional sine calibration. Non-planar bending tests qualitatively demonstrate the sensor’s three-dimensional reconstruction capability; accuracy along the complete 3D centerline remains unverified. The results demonstrate that improved calibration accuracy can be achieved with the demonstrated single-rotation-stage groove fixture by applying the proposed correction, without requiring local angular feedback or synchronized dual-end rotation.

## Figures and Tables

**Table 1:** Coefficients of determination (*R*^2^) for sinusoidal fits of sensor plane 10 using conventional sine calibration and rotation-angle correction.

R-Squares of Sinusoidal Fit from Sensor Plane 10 with Different Calibration Methods				
Calibration method	FBG_1,10_	FBG_2,10_	FBG_3,10_	FBG_4,10_
Conventional sine calibration	0.9570	0.9517	0.9518	0.9576
Rotation-angle correction	0.9999	0.9999	0.9998	0.9999

**Table 2:** Endpoint-position errors for the 50-plane sensor. Each target has five records per method; all errors are in millimeters. A denotes rotation-angle correction and B denotes conventional sine calibration. SD(*n* − 1) is the sample standard deviation across all records in the dataset.

Dataset	Records/ Targets	Mean [mm]	RMSE [mm]	SD(*n* − 1) [mm]	Min. [mm]	Max. [mm]
50-A	25/5	11.28	13.73	7.98	0.29	23.02
50-B	25/5	19.03	21.55	10.31	2.39	34.41

RMSE, root-mean-square error.

**Table 3:** Comparison of representative MCF-based shape-sensing studies with the present work.

Study	Sensor Configuration	Sensor Length	No. of FBGs	FBG Spacing	Validation Shape	Reconstruction Error	Remarks
Bronnikov 2019 [23]	7-core MCF; 4 cores used	72 mm	24	14 mm	Planar arcs and 3D spirals	Mean maximum absolute error: 0.85–1.57 mm; mean maximum relative error: 1.89–2.18%.	A minimum radius of 2.6 mm was demonstrated with a separate single-node sensor.
Khan 2019 [13]	4 × 4-core MCFs	118 mm	96	N/R	8 planar/3D catheter shapes	Max. error 1.05 mm; max. mean 0.44 mm	Multi-fiber catheter configuration
Idrisov 2021 [19]	7-core MCF	72 mm	56	N/R	Planar; R = 40–70 mm	Rel. curvature error: avg. 0.50%; max. 1.37%	Calibration focused
Fu 2024 [15]	7-core MCF; WFBG	600 mm	420	8 mm Gap	2D/3D shapes	Min. error/length: 1.08% (2D); 1.07% (3D)	OFDR/WFBG sampling
Meng 2024 [17]	7-core MCF; OFDR	730–1090 mm	N/A	N/A	Semicircular grooves	Max. rel. end-position error 3.37%; temp. max. 5.1%	Distributed OFDR
This work	4-core MCF	200 mm	200	4 mm	Planar R = 50–200 mm; endpoint; qualitative 3D	Tip error: ≈2–5 mm (planar); endpoint mean 11.28 mm (rotation-angle correction) vs. 19.03 mm (sine)	Single-rotation-stage groove calibration

Abbreviations: FBG, fiber Bragg grating; MCF, multicore fiber; WFBG, weak fiber Bragg grating; OFDR, optical frequency-domain reflectometry; N/R, not reported; N/A, not applicable; 2D, two-dimensional; 3D, three-dimensional. R denotes the bending radius.

**Figure A1:**
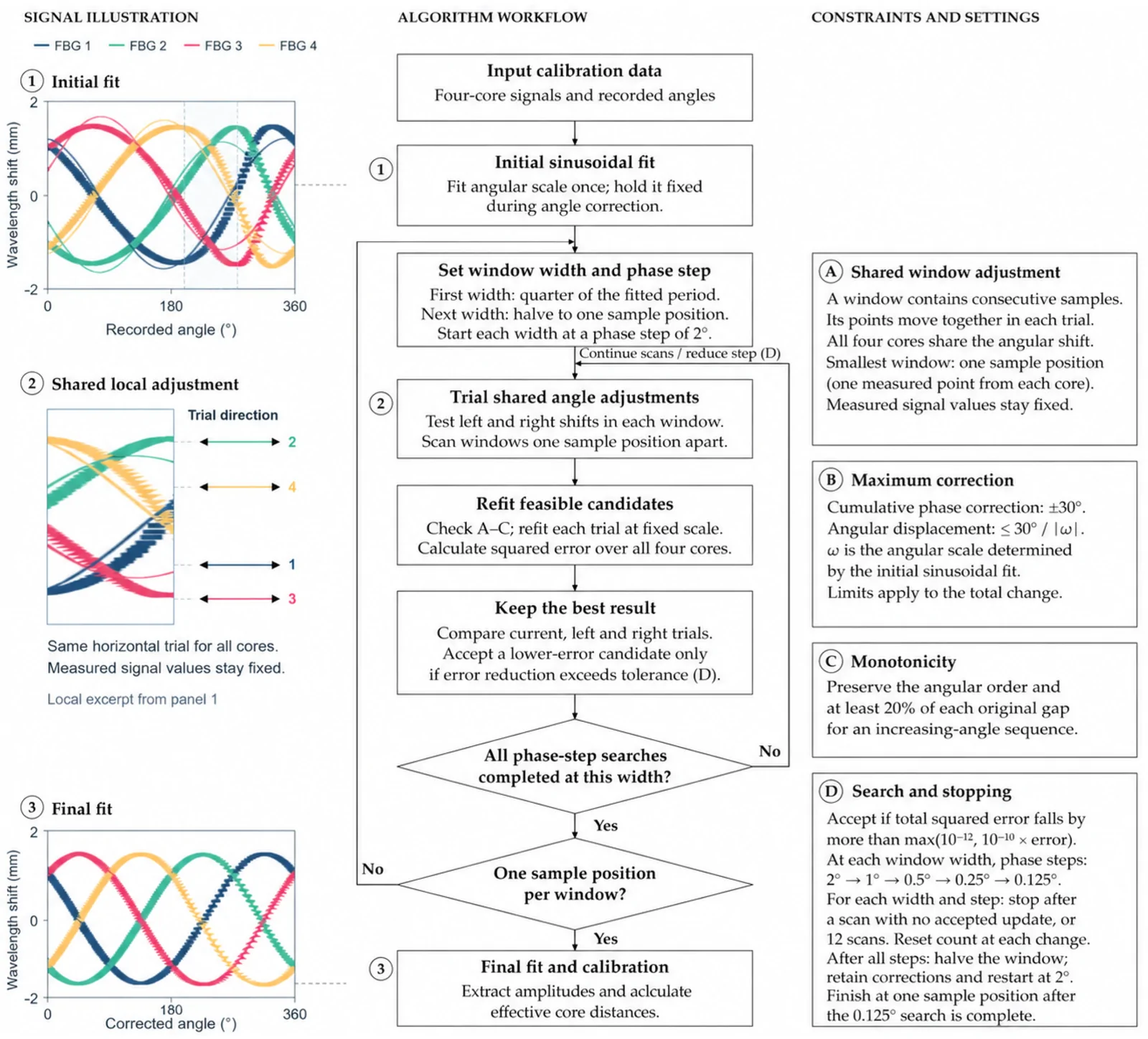
Shared angle correction of the four FBG responses. Panels 1 and 3 show excerpts from Figure 5 before and after correction. Panel 2 enlarges the highlighted interval; its arrows indicate trial directions, not measured displacements or recorded intermediate iterations. Boxes A–D specify the constraints and search settings.

**Figure A6:**
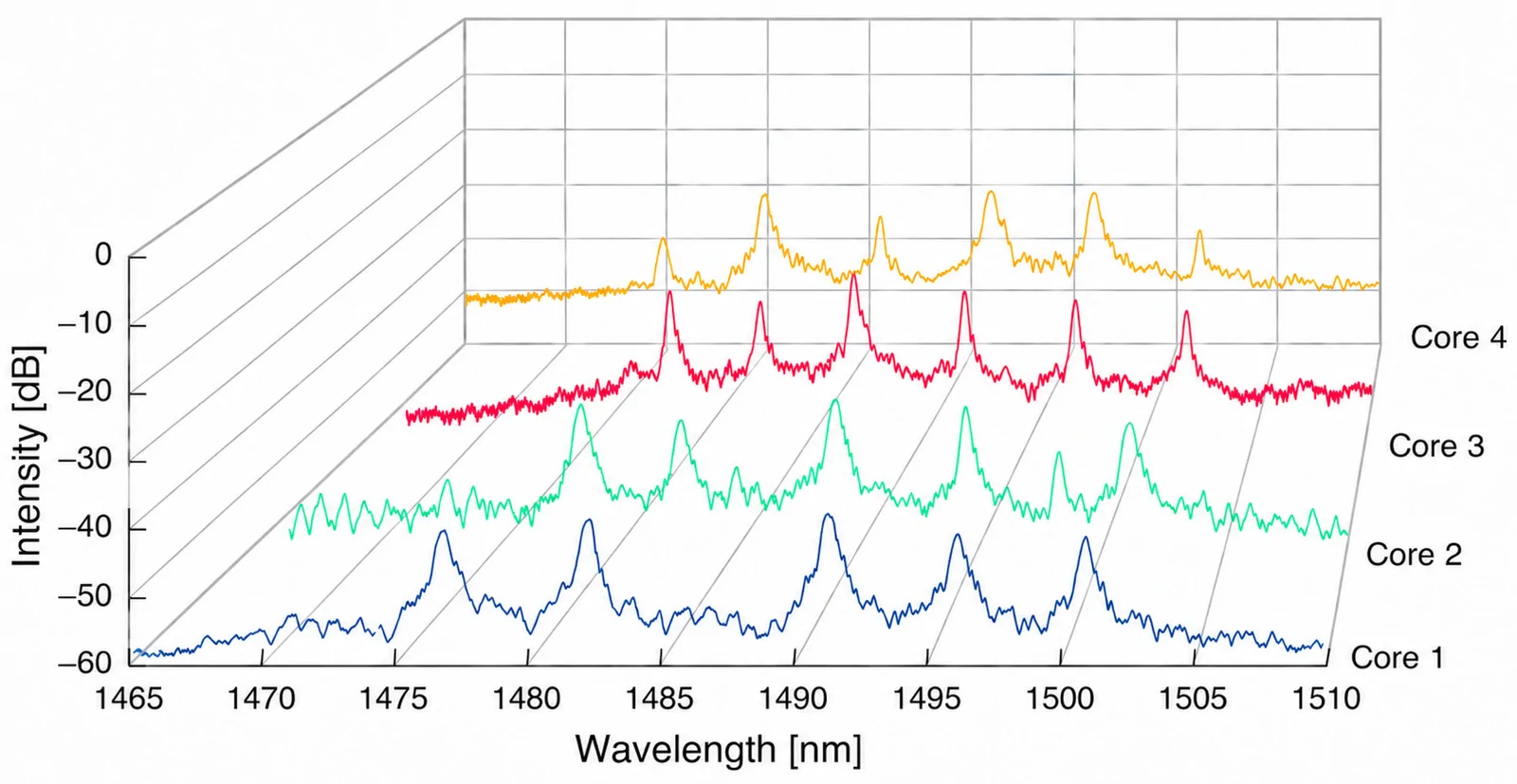
Reflection spectra of the independently fabricated sensor with 5 sensor planes.

**Figure A7:**
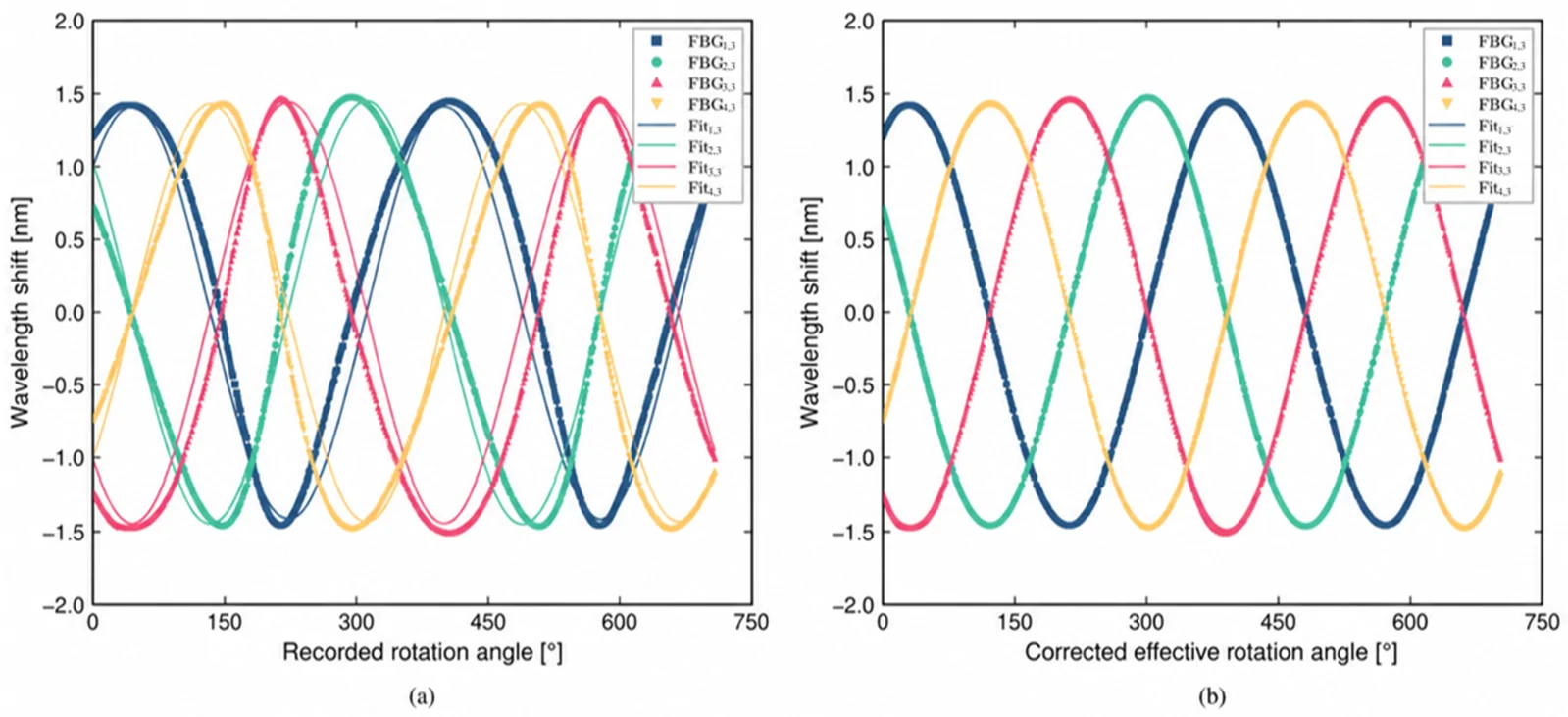
Four-core responses and sinusoidal fits for the 5-plane sensor using (**a**) conventional sine calibration and (**b**) rotation-angle correction.

**Figure A8:**
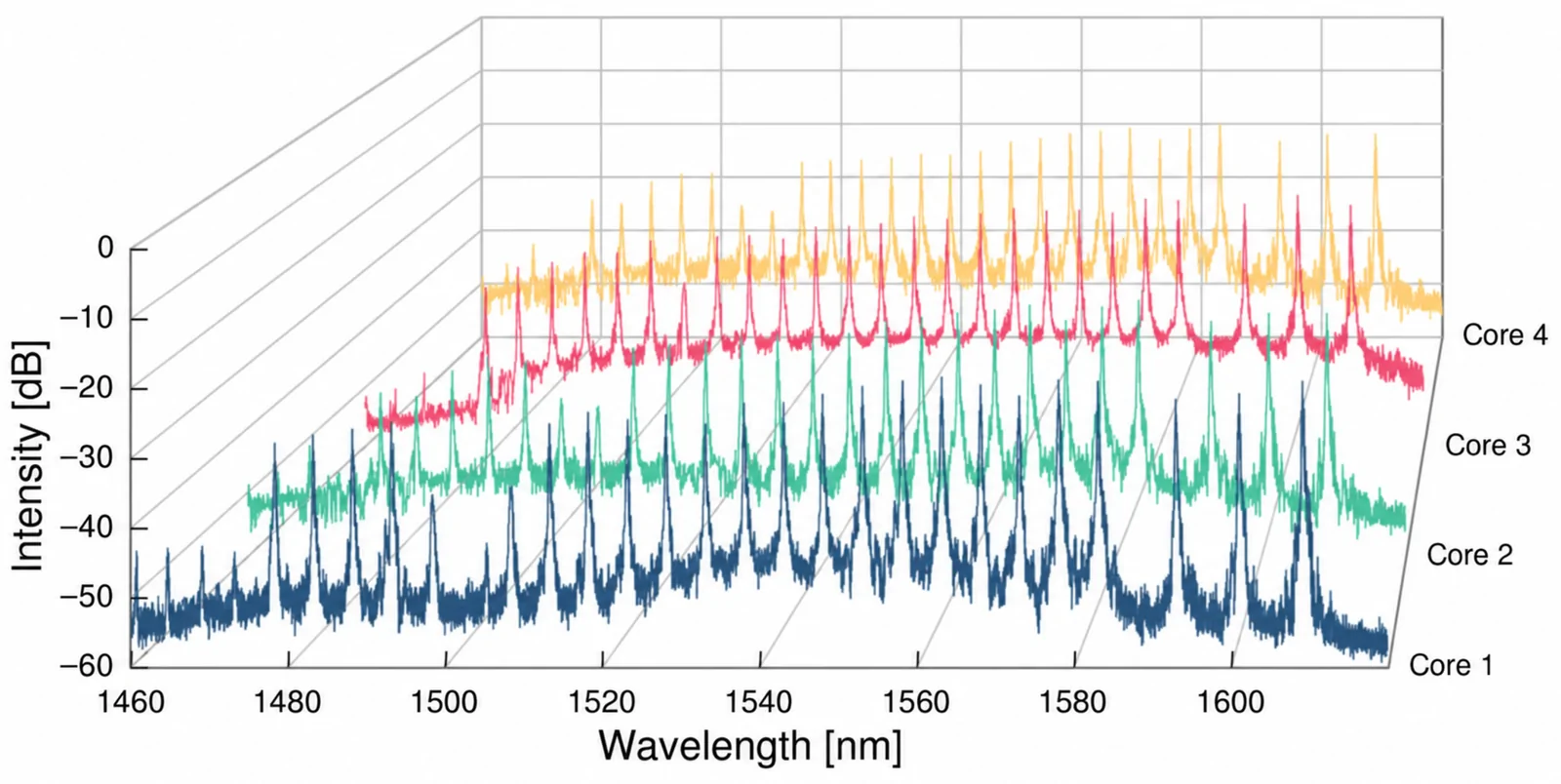
Reflection spectra of the independently fabricated sensor with 25 sensor planes.

**Figure A9:**
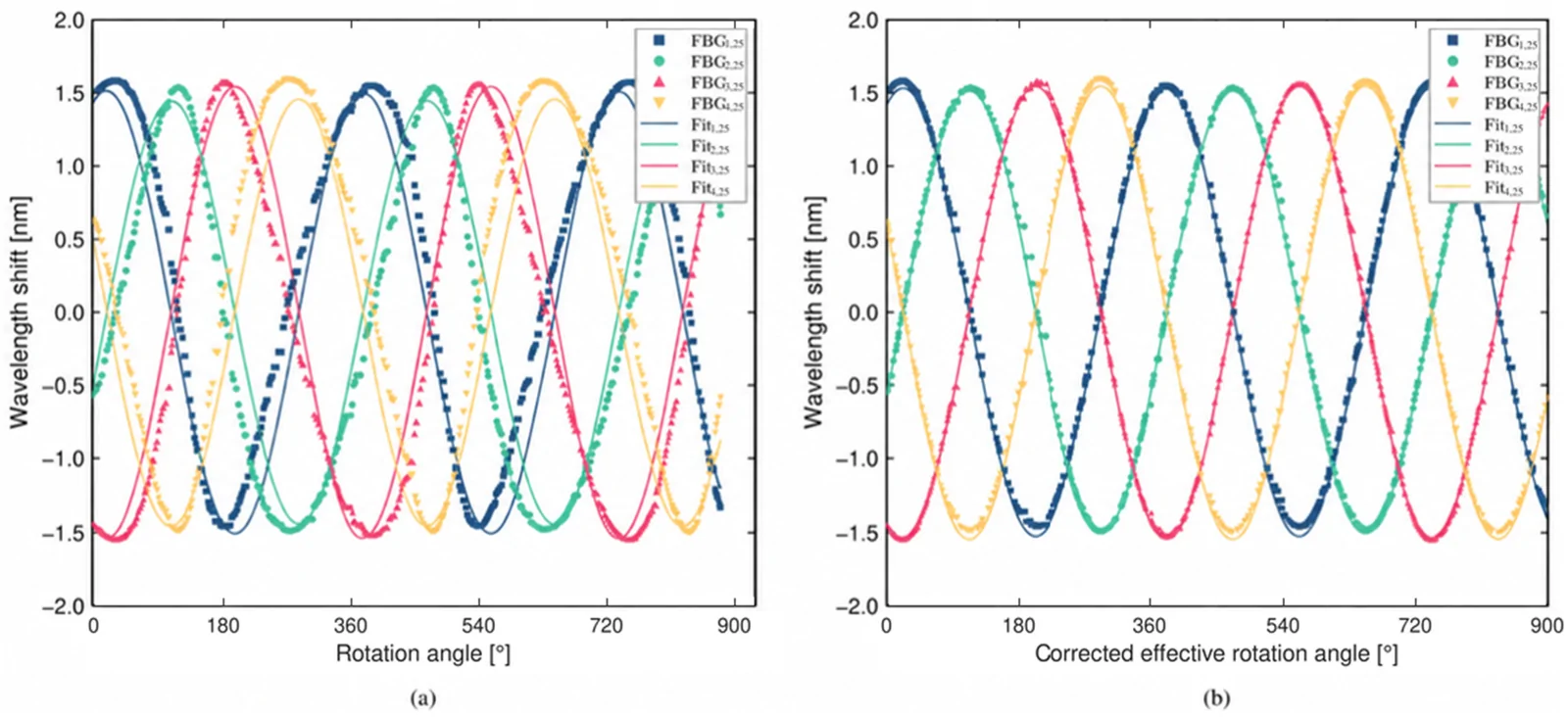
Four-core responses and sinusoidal fits for the 25-plane sensor using (**a**) conventional sine calibration and (**b**) rotation-angle correction.

## Data Availability

The data presented in this study are available from the corresponding author upon reasonable request.

## Appendix A. Angular Correction Procedure

Figure A1 summarizes the correction applied separately to each sensor plane. At each rotation sample position, the four FBG responses share the same angular correction, while their measured wavelength shifts remain unchanged. The search progressively narrows a moving window along the rotation sweep, ending with one sample position containing four measured points. The left-hand panels illustrate the main stages using sensor plane 10; the center shows the workflow, and boxes A–D give the constraints and search settings.

Initial fit. The four FBG responses are first fitted against the recorded rotation angle using the Levenberg–Marquardt method described in Section 4. This fit determines the initial amplitudes, offsets and phase, together with the angular scaling parameter *ω*. The angular scale is then held fixed during angle correction, so that subsequent adjustments describe local departures from the initial fit.

Shared window adjustment (A). A window contains consecutive sample positions along the rotation sweep of one sensor plane. Its initial width is approximately one quarter of the period obtained from the initial fit, expressed as a whole number of sample positions and rounded up. The procedure starts with the first window, tests a shared leftward and rightward angular shift, and advances the window by one sample position at a time until the last full window reaches the end of the data. This constitutes one scan. All points in the current window receive the same trial shift, including the four cores at every sample position; measured wavelength shifts stay fixed. Windows overlap, so a point can be adjusted again in later trials. As the window is narrowed, the final width is one sample position, at which the angles assigned to its four core responses are adjusted together. Panel 2 illustrates the shared horizontal trial direction.

Correction limits and angular order (B–C). The cumulative correction is limited to ±30° in model phase, corresponding to a maximum angular displacement of 30°/|*ω*| from the recorded angle. This bound applies to the accumulated correction throughout the search and is not reset when the window width or step size changes. For an increasing-angle sequence, corrected angles must retain their original order and at least 20% of every original angular gap, including the gaps at the window boundaries. Trials that violate these conditions are discarded.

Candidate comparison. Each feasible trial is refitted with the angular scale held fixed. The fitting error is the sum of squared residuals over the complete four-core responses of the sensor plane, not only the points in the current window. The current result and the left- and right-shifted trials are compared, and a lower-error candidate is accepted only when the error decrease exceeds the tolerance in box D. Otherwise, the current angles are retained. The window then advances to the next sample position.

Search resolution and stopping (D). For each fixed window width, the trial phase step is reduced successively from 2° to 1°, 0.5°, 0.25° and 0.125°. At each step size, scans continue until a full scan produces no accepted update or 12 scans have been completed, whichever occurs first. The scan count is reset for each new step size. After all five step sizes have been tested, the window width is halved, rounding up to a whole number of sample positions, and the phase step restarts at 2°. The corrected angles are retained, and the scan count is reset. The entire search ends only after the 0.125° search has finished for a window containing one sample position.

An update is accepted only if the decrease in total squared fitting error exceeds the larger of 10^−12^ and 10^−10^ times the current error. This tolerance avoids accepting changes that are too small to be numerically meaningful. The correction bound and trial steps are specified in the model phase. Dividing these phase increments by |*ω*| gives the corresponding shifts in the recorded rotation angles.

Final fit and calibration. The corrected angles are held fixed for the final sinusoidal fit. The resulting amplitudes are converted into effective FBG-to-neutral-axis distances using Equation (19). Figure A1 illustrates the fitting procedure; its calibration benefit is assessed by the bending-radius and shape-reconstruction results in Figure 6 and Section 5. The physical interpretation of the corrected angles is discussed in Section 6.2.

## Appendix B. Full-Array Fitting Statistics and Effective Core Distances

Figure A2, Figure A3, Figure A4 and Figure A5 compare conventional sine calibration and rotation-angle correction for sensor planes 20, 30, 40 and 50. Both methods use the same measured wavelength shifts, plotted against the recorded and corrected effective rotation angles, respectively.

Table A1 summarizes the sinusoidal-fit R^2^ values for all 50 sensor planes. For each plane, the mean, sample standard deviation (SD), minimum and maximum are calculated across its four FBG responses; the overall row includes all 200 responses. Across the array, the R^2^ range changes from 0.9502–0.9981 with conventional sine calibration to 0.9855–0.9999 with rotation-angle correction. These statistics quantify in-sample agreement with the sinusoidal model rather than local-angle or bending-radius accuracy.

Table A2 summarizes the effective FBG-to-neutral-axis distances obtained after rotation-angle correction using Equation (19). For each core, N denotes the number of sensor planes included in the statistics (N = 50). The sample SD describes variation across planes rather than uncertainty from repeated calibration. In both tables, sample SD is calculated using one fewer than the number of observations in the denominator. The distances are effective calibration parameters, not opposite-core separations or independently measured physical core positions. The separate bending-radius assessment in Figure 6 is limited to sensor plane 10.

**Table A1:** Sinusoidal-fit statistics for all 50 sensor planes before and after rotation-angle correction.

Sensor Plane	Conventional Sine Calibration R^2^ Mean ± SD	Rotation-Angle Correction R^2^ Mean ± SD	Conventional R^2^ Min.–Max.	Corrected R^2^ Min.–Max.
1	0.99530 ± 0.00028	0.99980 ± 0.00000	0.9949–0.9955	0.9998–0.9998
2	0.99393 ± 0.00045	0.99948 ± 0.00010	0.9933–0.9943	0.9994–0.9996
3	0.98505 ± 0.00097	0.99913 ± 0.00015	0.9838–0.9860	0.9990–0.9993
4	0.97755 ± 0.00175	0.99878 ± 0.00026	0.9750–0.9790	0.9985–0.9990
5	0.99675 ± 0.00013	0.99965 ± 0.00006	0.9966–0.9969	0.9996–0.9997
6	0.99685 ± 0.00037	0.99895 ± 0.00013	0.9964–0.9973	0.9988–0.9991
7	0.99603 ± 0.00043	0.99963 ± 0.00013	0.9954–0.9964	0.9995–0.9998
8	0.99223 ± 0.00071	0.99948 ± 0.00010	0.9914–0.9931	0.9994–0.9996
9	0.99670 ± 0.00024	0.99935 ± 0.00010	0.9965–0.9970	0.9992–0.9994
10	0.95453 ± 0.00321	0.99988 ± 0.00005	0.9517–0.9576	0.9998–0.9999
11	0.97078 ± 0.00334	0.99323 ± 0.00339	0.9658–0.9729	0.9890–0.9970
12	0.98550 ± 0.00161	0.99943 ± 0.00017	0.9831–0.9865	0.9992–0.9996
13	0.99668 ± 0.00021	0.99940 ± 0.00008	0.9964–0.9969	0.9993–0.9995
14	0.97875 ± 0.00101	0.99650 ± 0.00105	0.9779–0.9802	0.9952–0.9974
15	0.99680 ± 0.00038	0.99923 ± 0.00013	0.9963–0.9971	0.9991–0.9994
16	0.97913 ± 0.00230	0.99378 ± 0.00206	0.9766–0.9821	0.9918–0.9957
17	0.97915 ± 0.00166	0.99385 ± 0.00240	0.9773–0.9806	0.9913–0.9960
18	0.97680 ± 0.00076	0.99643 ± 0.00065	0.9761–0.9776	0.9958–0.9973
19	0.97280 ± 0.00165	0.99648 ± 0.00088	0.9705–0.9744	0.9952–0.9971
20	0.99803 ± 0.00005	0.99965 ± 0.00006	0.9980–0.9981	0.9996–0.9997
21	0.96373 ± 0.00311	0.99808 ± 0.00046	0.9614–0.9683	0.9974–0.9984
22	0.99403 ± 0.00046	0.99958 ± 0.00010	0.9937–0.9947	0.9995–0.9997
23	0.98790 ± 0.00034	0.99953 ± 0.00010	0.9877–0.9884	0.9994–0.9996
24	0.97558 ± 0.00209	0.99613 ± 0.00088	0.9734–0.9776	0.9954–0.9973
25	0.98790 ± 0.00091	0.99915 ± 0.00010	0.9868–0.9889	0.9991–0.9993
26	0.99443 ± 0.00051	0.99925 ± 0.00026	0.9940–0.9950	0.9989–0.9995
27	0.99525 ± 0.00050	0.99905 ± 0.00031	0.9946–0.9958	0.9987–0.9994
28	0.99140 ± 0.00078	0.99953 ± 0.00010	0.9904–0.9922	0.9994–0.9996
29	0.96033 ± 0.00381	0.99838 ± 0.00030	0.9571–0.9652	0.9981–0.9988
30	0.96798 ± 0.00212	0.99500 ± 0.00157	0.9659–0.9700	0.9934–0.9964
31	0.99583 ± 0.00030	0.99918 ± 0.00025	0.9955–0.9962	0.9988–0.9993
32	0.99703 ± 0.00043	0.99960 ± 0.00008	0.9967–0.9976	0.9995–0.9997
33	0.98488 ± 0.00162	0.99485 ± 0.00127	0.9832–0.9870	0.9938–0.9967
34	0.97470 ± 0.00266	0.99835 ± 0.00052	0.9713–0.9771	0.9977–0.9989
35	0.99625 ± 0.00024	0.99960 ± 0.00000	0.9960–0.9965	0.9996–0.9996
36	0.99135 ± 0.00024	0.99893 ± 0.00015	0.9911–0.9916	0.9988–0.9991
37	0.97545 ± 0.00033	0.99743 ± 0.00048	0.9750–0.9757	0.9970–0.9981
38	0.99603 ± 0.00021	0.99853 ± 0.00031	0.9958–0.9963	0.9981–0.9988
39	0.99258 ± 0.00030	0.99873 ± 0.00033	0.9922–0.9929	0.9984–0.9991
40	0.99023 ± 0.00091	0.99865 ± 0.00029	0.9891–0.9911	0.9984–0.9989
41	0.99643 ± 0.00022	0.99923 ± 0.00022	0.9962–0.9967	0.9989–0.9994
42	0.99710 ± 0.00026	0.99968 ± 0.00010	0.9968–0.9974	0.9996–0.9998
43	0.99310 ± 0.00061	0.99880 ± 0.00036	0.9923–0.9937	0.9983–0.9991
44	0.99325 ± 0.00062	0.99940 ± 0.00000	0.9927–0.9941	0.9994–0.9994
45	0.97725 ± 0.00100	0.99738 ± 0.00079	0.9765–0.9787	0.9962–0.9979
46	0.99705 ± 0.00025	0.99968 ± 0.00005	0.9967–0.9973	0.9996–0.9997
47	0.95670 ± 0.00184	0.99905 ± 0.00019	0.9545–0.9590	0.9988–0.9992
48	0.96805 ± 0.00289	0.99810 ± 0.00035	0.9647–0.9714	0.9976–0.9984
49	0.99533 ± 0.00022	0.99828 ± 0.00026	0.9951–0.9956	0.9979–0.9985
50	0.95338 ± 0.00292	0.98850 ± 0.00318	0.9502–0.9561	0.9855–0.9913
All 200	0.98519 ± 0.01286	0.99815 ± 0.00232	0.9502–0.9981	0.9855–0.9999

N, number of sensor planes; SD, sample standard deviation across the 50 sensor planes for each core.

**Table A2:** Effective core-distance statistics after rotation-angle correction across all 50 sensor planes.

Core	N	Mean Effective Distance [μm]	SD [μm]	Min. [μm]	Max. [μm]
Core 1	50	39.384	0.221	38.884	39.859
Core 2	50	39.265	0.217	38.685	39.714
Core 3	50	38.036	0.196	37.498	38.497
Core 4	50	38.103	0.230	37.521	38.675

N, number of sensor planes; SD, sample standard deviation across the 50 sensor planes for each core.

## Appendix C. Additional Independently Fabricated Sensors

Two additional sensors containing 5 and 25 sensor planes were fabricated and calibrated independently of the 50-plane sensor. Figure A6, Figure A7, Figure A8 and Figure A9 and Table A3 and Table A4 present their spectra and calibration examples using the rotation-angle correction described in Appendix A. Each fabricated sensor and each sensor plane requires independent calibration of its effective FBG distances and angular orientation; the calibration parameter values are not transferred between sensors.

### Appendix C.1. Sensor with 5 Planes

**Table A3:** Coefficients of determination (R^2^) for the calibration example of the 5-plane sensor.

Calibration Method	FBG_1,3_	FBG_2,3_	FBG_3,3_	FBG_4,3_
Conventional sine calibration	0.9500	0.9729	0.9417	0.9693
Rotation-angle correction	0.9997	0.9998	0.9997	0.9994

### Appendix C.2. Sensor with 25 Planes

**Table A4:** Coefficients of determination (R^2^) for the calibration example of the 25-plane sensor.

Calibration Method	FBG_1,25_	FBG_2,25_	FBG_3,25_	FBG_4,25_
Conventional sine calibration	0.9358	0.9436	0.9561	0.9186
Rotation-angle correction	0.9979	0.9987	0.9991	0.9983
